# Nuclear receptor Nur77 resolves the inflammatory response of macrophages primarily by regulating expression of AP-1 transcription factors and their target genes

**DOI:** 10.1093/nar/gkag808

**Published:** 2026-08-17

**Authors:** Sanne C Lith, Sebastian Gregoricchio, Claudia M van Tiel, Ingeborg van der Made, Beatriz M Freire, Simon Linder, Sanne Jacobs, Onno Bleijerveld, Liesbeth Hoekman, Guglielmo L Alonzo, Wilbert Zwart, Marten A Hoeksema, Carlie J M de Vries

**Affiliations:** Amsterdam UMC location, location Academic Medical Center, Department of Medical Biochemistry, University of Amsterdam, 1105AZ Amsterdam, The Netherlands; Amsterdam Cardiovascular Sciences, University of Amsterdam, 1105AZ Amsterdam, The Netherlands; Amsterdam Institute for Immunology and Infectious Diseases, University of Amsterdam, 1105AZ Amsterdam, The Netherlands; Division of Oncogenomics, Netherlands Cancer Institute and Oncode Institute, 1066CX Amsterdam, The Netherlands; Amsterdam UMC location, location Academic Medical Center, Department of Medical Biochemistry, University of Amsterdam, 1105AZ Amsterdam, The Netherlands; Amsterdam Cardiovascular Sciences, University of Amsterdam, 1105AZ Amsterdam, The Netherlands; Amsterdam Institute for Immunology and Infectious Diseases, University of Amsterdam, 1105AZ Amsterdam, The Netherlands; Amsterdam UMC location, location Academic Medical Center, Department of Medical Biochemistry, University of Amsterdam, 1105AZ Amsterdam, The Netherlands; Amsterdam Cardiovascular Sciences, University of Amsterdam, 1105AZ Amsterdam, The Netherlands; Amsterdam Institute for Immunology and Infectious Diseases, University of Amsterdam, 1105AZ Amsterdam, The Netherlands; Amsterdam UMC location, location Academic Medical Center, Department of Medical Biochemistry, University of Amsterdam, 1105AZ Amsterdam, The Netherlands; Amsterdam Cardiovascular Sciences, University of Amsterdam, 1105AZ Amsterdam, The Netherlands; Amsterdam Institute for Immunology and Infectious Diseases, University of Amsterdam, 1105AZ Amsterdam, The Netherlands; Division of Oncogenomics, Netherlands Cancer Institute and Oncode Institute, 1066CX Amsterdam, The Netherlands; Amsterdam UMC location, location Academic Medical Center, Department of Medical Biochemistry, University of Amsterdam, 1105AZ Amsterdam, The Netherlands; Amsterdam Cardiovascular Sciences, University of Amsterdam, 1105AZ Amsterdam, The Netherlands; Amsterdam Institute for Immunology and Infectious Diseases, University of Amsterdam, 1105AZ Amsterdam, The Netherlands; Mass Spectrometry/Proteomics Facility, Netherlands Cancer Institute, 1066CX Amsterdam, The Netherlands; Mass Spectrometry/Proteomics Facility, Netherlands Cancer Institute, 1066CX Amsterdam, The Netherlands; Amsterdam UMC location, location Academic Medical Center, Department of Medical Biochemistry, University of Amsterdam, 1105AZ Amsterdam, The Netherlands; Amsterdam Cardiovascular Sciences, University of Amsterdam, 1105AZ Amsterdam, The Netherlands; Amsterdam Institute for Immunology and Infectious Diseases, University of Amsterdam, 1105AZ Amsterdam, The Netherlands; Division of Oncogenomics, Netherlands Cancer Institute and Oncode Institute, 1066CX Amsterdam, The Netherlands; Amsterdam UMC location, location Academic Medical Center, Department of Medical Biochemistry, University of Amsterdam, 1105AZ Amsterdam, The Netherlands; Amsterdam Cardiovascular Sciences, University of Amsterdam, 1105AZ Amsterdam, The Netherlands; Amsterdam Institute for Immunology and Infectious Diseases, University of Amsterdam, 1105AZ Amsterdam, The Netherlands; Amsterdam UMC location, location Academic Medical Center, Department of Medical Biochemistry, University of Amsterdam, 1105AZ Amsterdam, The Netherlands; Amsterdam Cardiovascular Sciences, University of Amsterdam, 1105AZ Amsterdam, The Netherlands; Amsterdam Institute for Immunology and Infectious Diseases, University of Amsterdam, 1105AZ Amsterdam, The Netherlands

## Abstract

The nuclear receptor Nur77 plays a crucial, protective role in chronic inflammatory diseases and deficiency of Nur77 in macrophages results in excessive pro-inflammatory cytokine secretion. Previous research suggested that Nur77’s regulatory function in inflammation is due to repression of the pro-inflammatory transcription factor NF-kB, but the underlying mechanism remains unclear. To address this, we applied a genome-wide, multi-omics approach in LPS-stimulated RAW264.7 macrophages with inducible Nur77 expression. Key findings were validated in wild-type and Nur77-deficient bone marrow-derived macrophages. We show that Nur77 suppresses the expression of inflammatory genes through a dual mechanism wherein Nur77 acts as a repressor of AP-1 targets at two levels: first, Nur77 occupies regulatory elements proximal to AP-1 target genes through AP-1 motifs and second, Nur77 regulates the expression of AP-1 family members themselves. These repressive activities of Nur77 result in diminished RNA Pol II on AP-1 genes and their targets. The first zinc finger of the Nur77 DNA-binding domain is required to reduce AP-1 activity. In summary, Nur77 represses macrophage inflammation through regulation of both immediate-early AP-1 expression, as well as inhibition of AP-1-driven gene programs.

## Graphical Abstract

**Graphical Abstract:**
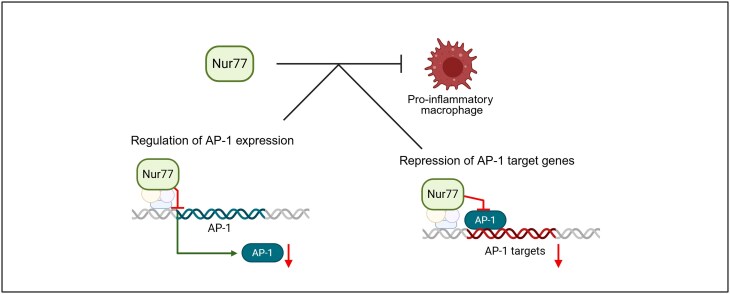

## Introduction

Macrophages are distributed throughout the body and pivotal for tissue homeostasis and adequate immune responses. Tissue-resident macrophages originate from the embryonic early yolk sac or fetal liver, whereas macrophages that are recruited upon acute inflammation differentiate from circulating, bone marrow-derived monocytes [1]. During an immune response, the multifaceted role of macrophages includes induction of inflammation, as well as control of inflammation, and initiation of tissue repair [2]. As a consequence, regulating macrophage activity is essential for maintaining the delicate balance of sufficient immune response without causing severe tissue damage [3]. The macrophage phenotype can be broadly categorized in pro-inflammation or pro-resolution. This is dictated by a variety of clues present in the microenvironment surrounding these immune cells, such as the inerrant characteristics of the tissue, the class of pathogens (i.e. which pattern-recognition receptor will be activated), and cytokines secreted by other immune cells [4]. Pro-inflammatory macrophage phenotypes have been implicated in contributing to chronic inflammatory diseases such as atherosclerosis, rheumatoid arthritis, and inflammatory bowel disease [5–7]. These findings underscore the importance of understanding the biology of macrophage function and identifying the key regulatory factors involved in balancing these immune cells’ pro- and anti-inflammatory impact.

Typically, to study the inflammatory response of monocyte-derived macrophages *in vitro*, lipopolysaccharide (LPS) is used to bind Toll-like receptor 4 (TLR4), triggering a cascade of intracellular signaling events that govern a pro-inflammatory program [8]. As a consequence, the expression and activity of immediate-early genes is initiated. Upstream regulators of these genes include transcription factors such as NF-κB, CREB, and SRF, which become active through protein phosphorylation and cellular translocation events without the need for new protein synthesis [9]. Immediate-early genes are typically characterized by their rapid induction, occurring within minutes of stimulation, and their transient nature. Many of the immediate-early genes belong to the activator protein-1 (AP-1) family of proteins that comprises transcription factors of the Jun (JunB, JunD), Fos (FRA-1/2, FosB), Maf, and ATF subfamilies [10]. In macrophages, immediate-early genes are crucial to drive the subsequent expression of pro-inflammatory cytokines and are subject to regulation by the MAPK pathway [11, 12].

Nur77, also known as NR4A1, is a nuclear receptor implicated in several cellular processes, such as cell survival, apoptosis, metabolism, and the immune system [13]. This transcription factor fulfills a noticeable anti-inflammatory role across various immune cells, including T cells, dendritic cells, and macrophages [14–17]. The expression of Nur77 is rapidly induced in macrophages and dendritic cells in response to inflammatory stimuli, and in T cells upon activation of T cell receptors [14, 18]. In mice, the protective function of Nur77 has been studied in multiple chronic inflammatory disease models, consistently demonstrating that Nur77 deficiency leads to aggravation of atherosclerosis, inflammatory bowel disease, and multiple sclerosis [19–22]. The impact of Nur77 on macrophage function concerns several aspects. It has been shown that Nur77-deficient mice lack a sub-population of circulating monocytes, the so-called Ly6C^lo^ monocytes, which monitor the integrity of the vascular endothelial barrier [23–25]. In addition, Nur77-mediated reduction of the inflammatory phenotype of activated macrophages involves reprogramming of mitochondrial metabolism [26]. Similar to other nuclear receptors such as the glucocorticoid receptor, Nur77 can repress the pro-inflammatory transcription factor NF-kB [26, 27]. Despite these insights, the exact signaling pathways through which Nur77 operates to modulate pro-inflammatory macrophages and its role during the resolution phase of inflammation remain elusive.

In this study, we investigate the impact of Nur77 on the inflammatory response of macrophages, utilizing a Nur77-inducible overexpression model in RAW264.7 macrophage (RAW-Nur77) cells and bone marrow-derived macrophages that are either wild type (BMDM-WT) or deficient (BMDM-Nur77-KO) for Nur77. Employing multiple omics analyses, we elucidate the influence of Nur77 on inflammatory gene expression, with or without LPS exposure. Transcriptomics (RNA-seq) analyses confirmed that Nur77 suppresses the expression of AP-1 targets, resulting in a shift toward a less pro-inflammatory phenotype of activated macrophages. Integration of assay for transposase-accessible chromatin sequencing (ATAC-seq), chromatin immunoprecipitation sequencing (ChIP-seq), and rapid immunoprecipitation mass spectrometry of endogenous protein (RIME) datastreams revealed that Nur77 diminishes RNA Polymerase II (Pol II) binding, H3K27 acetylation, and DNA accessibility at AP-1 target genes. In addition, Nur77 exerts a regulatory effect on the transcription of AP-1 factors. To substantiate the importance of protein–protein interactions for Nur77 to execute its function, we demonstrated in split-luciferase assays that Nur77 interacts with cJun and cFos. Interestingly, Nur77-variants lacking the first zinc finger (Nur77-∆Znf1) or with a two amino-acid mutation in this zinc finger (Nur77-CE-mutant), thus losing DNA-binding capacity, no longer inhibit AP-1 activity and lose their anti-inflammatory proficiency. In conclusion, Nur77 dampens the proinflammatory response of macrophages by affecting AP-1 target genes at two levels: by regulating expression of AP-1 genes and as an inhibitor of AP-1 target genes.

## Materials and methods

### *In vitro* cell lines

The mouse macrophage RAW264.7 cell line was cultured in RPMI medium (Gibco, cat#22400089) supplemented with 10% fetal calf serum, 100 U/ml penicillin, 100 µg/ml streptomycin, and 2 mM l-glutamine. The RAW264.7 cell line with doxycycline-inducible overexpression of green fluorescent protein (GFP) (RAW-Ctrl), Nur77-HA (RAW-Nur77), an HA-epitope tagged Nur77-mutant lacking the first zinc finger (ΔZnf1-HA), or an HA-epitope tagged Nur77-mutant with a mutation in the first zinc finger (Nur77-CE284-285AA (Nur77-CE-mutant-HA) was cultured as described before [26]. Human embryonic kidney (HEK) 293T cells were cultured in Dulbecco’s modified Eagle’s medium (DMEM; Gibco, cat#31885023) containing the supplements 10% fetal calf serum, 100 µg/ml streptomycin, 100 U/ml penicillin, and 10 mM l-glutamine.

### Mouse strains

All animal housing, care, and experimental procedures were conducted in compliance with the guidelines and regulations established by the Institutional Animal Experimental Committee for Animal Welfare, adhering to the principles of Directive 2010/63/EU of the European Parliament. Whole-body Nur77 knockout mice were generated by breeding C57Bl6/J mice that overexpress Cre recombinase under the control of the human cytomegalovirus (CMV) promoter with mice carrying a loxP-flanked Nur77 (Nr4a1) locus [28]. This breeding strategy allowed for the deletion of the Nur77 gene. Successful deletion was confirmed through genotyping polymerase chain reaction (PCR). Subsequently, Nur77-deficient mice lacking Cre recombinase expression were produced (referred to as Nur77-KO mice). Both Nur77-KO mice and their wild-type (WT) littermates were used in the experiments.

### Bone marrow-derived macrophage differentiation and cell culture

Bone marrow cells were harvested from femurs and tibias of male WT and Nur77-KO mice aged 6–12 weeks. These cells were then differentiated into macrophages by culturing them in RPMI 1640 medium supplemented with 10% fetal calf serum, 100 U/ml penicillin, 100 μg/ml streptomycin, 2 mM l-glutamine, 15% L929-conditioned medium, 1 mM sodium pyruvate, 1× MEM non-essential amino acids solution (Gibco, cat#1140050), and 1× MEM vitamin solution (Gibco, cat#11120052) for a period of 7 days. BMDMs were then seeded at a density of 1 × 10^6^ cells per well in six-well culture plates. Post differentiation, cells were lifted, counted, and re-seeded for subsequent analyses. For experiments involving LPS-stimulated BMDMs, 100 ng/ml of lipopolysaccharide (LPS) (Sigma, cat#L2637) was used. To confirm successful differentiation, the expression of differentiation markers CD11b+ F4/80+ was assessed by flow cytometry, demonstrating that over >99% of cells were positive for both markers [29].

### Supernatant cytokine analysis

RAW-Ctrl and RAW-Nur77 were seeded at a density of 1 × 10^4^ in 96-well plates in four replicates. The next day, cells were treated with 50 ng/ml doxycycline (Sigma, cat#D9891) for 18 h, followed by a stimulation with 500 ng/ml LPS for 0 and 18 h. In the supernatant, cytokines were analyzed by flow cytometry utilizing the Legendplex kit (Biolegend, cat# 740150, lot# B406835) measuring IL-23, IL-1α, IFN-γ, TNF-α, CCL2, IL-12p70, IL-1β, IL-10, IL-6, IL-27, IL-17A, IFN-β, and GM-CSF secretion levels. Cytokine concentrations were determined using the Legendplex Data Analysis Software Suite.

### RNA-sequencing

#### Sample preparation and isolation

RAW-Ctrl and RAW-Nur77 were seeded at a density of 3 × 10^5^ in 12-well plates in four replicates. The next day, cells were treated with 50 ng/ml doxycycline for 18 h, followed by a stimulation with 500 ng/ml LPS for 0, 1, and 5 h. Wild-type and Nur77-deficient BMDMs were seeded at a density of 1 × 10^6^ cells in 12-well plates in four replicates and stimulated the next day with 100 ng/ml LPS for 0, 1, 5, and 18 h. RNA was extracted using the RNeasy Mini kit with an on-column DNase treatment according to the manufacturer’s instructions (Qiagen). RNA concentrations were determined using a Qubit fluorometer (Thermo) and RNA quality was evaluated using an Agilent Bioanalyzer 2100 system (Agilent Technologies).

#### Library preparation and NGS

Strand-specific libraries were generated using the KAPA mRNA HyperPrep kit (Roche, cat#8098123702) according to the manufacturer’s instructions. Libraries were pooled and diluted to 25 nM prior to sequencing on a NovaSeq 6000 instrument (Illumina) at a depth of 20 million paired-ended 150 base pair reads. Reads were aligned to the mouse genome mm10 using HISAT2 2.1.0 [30] with default settings.

#### Analysis of RNA-seq data

BAM files were indexed and filtered on MAPQ >15 with SAMtools 1.16.1 [31]. Raw tag counts and RPKM (reads per kilo base per million mapped reads) values per gene were summed using HOMER’s analyzeRepeats.pl script with default settings and the --noadj or --rpkm options for raw counts and RPKM reporting, respectively. Differential expression was assessed using the DESeq2 Bioconductor package in an R 4.2.2 environment with a Benjamini–Hochberg adjusted *P*-value <.05 and an average RPKM >1 in at least one group. Genes with a false discovery rate (FDR)-adjusted *P*-value <.05 and a log_2_FC > 1 or < -1 change were considered to be differentially regulated. Euler plots were generated with the R package eulerr (v7.0.0). Pathway analysis was performed using the software’s EGSEA [32] or Metascape [33]. Heat maps of gene expression values were created using the R package pheatmap (v1.0.12). Volcano plots of gene expression were created using the R package EnhancedVolcano (v1.18.0). Known motif analysis on the promoters of genes was performed using HOMER’s findMotifs.pl script with default settings.

### Chromatin immunoprecipitation sequencing

#### Sample preparation

RAW-Ctrl and RAW-Nur77 were seeded in T75 flasks at 3.75 × 10^6^ cells per flask. After 2 days, cells were treated with 50 ng/ml doxycycline for 18 h, followed by a stimulation with 500 ng/ml LPS for 0 and 1 h. Wild-type and Nur77-deficient BMDMs were seeded in T75 flasks at 15 × 10^6^ per flask and stimulated the next day with 100 ng/ml LPS for 0, 1, and 18 h. Cells were then washed twice with phosphate buffered saline (PBS) and only for HA-ChIP of RAW-Nur77 cells were cross-linked with 2 mM Di(N-succinimidyl) glutarate (Santa Cruz, cat#sc-285455B) in PBS for 30 min at room temperature. For all the ChIP targets, including HA-Nur77 cells, were then crosslinked with 1% formaldehyde (Thermo Fisher Scientific, cat#28908) for 10 min at room temperature. Crosslinking reactions were quenched by addition of glycine to 0.13 M final concentration and incubated for 2 min at room temperature. Cross-linked cells were washed twice with ice-cold PBS and collected by scraping in ice-cold PBS followed by centrifugation for 5 min at 800 × *g* and 4°C. Cell pellets were washed with ice-cold PBS followed by centrifugation for 5 min at 900 × *g* and 4°C. Cells were snap frozen and stored at −80°C until use. After thawing cell pellets on ice, cell membranes were lysed by resuspension in LB3 [10 mM Tris–HCl, pH 7.5, 100 mM NaCl, 1 mM ethylenediaminetetraacetic acid (EDTA), 0.5 mM EGTA, 0.1% Na-deoxycholate, 0.5% N-laurosylsarcosine] supplemented with protease inhibitor (Roche, cat#4693159001). Nuclei were ruptured and chromatin was sonicated into ~400 bp fragments using a Bioruptor Pico (Diagenode) for four cycles (double cross-linked) or three cycles (single cross-linked) of 10 s on and 50 s off. Sonicated samples were centrifuged for 10 min at 16 000 × *g* to pellet cell debris. Supernatants were transferred to DNA LoBind tubes (Sigma, cat#022431021) and diluted 1:10 with 10% Triton X-100 (Sigma, cat#T8787). Per ChIP reaction, 20 µl Protein A Dynabeads (Invitrogen, cat#10002D) together with 2 µl of HA (Diagenode, cat#C15200190), RNA polymerase (Diagenode, C1510055), or H3K27ac (Diagenode, C15410196) antibody were added to the chromatin sample and incubated overnight at 4°C. Immunoprecipitates were washed three times with wash buffer 1 [20 mM Tris–HCl, pH 7.4, 150 mM NaCl, 0.1% sodium dodecyl sulfate (SDS), 1% Triton X-100, 2 mM EDTA] supplemented with protease inhibitor, three times with wash buffer 3 (10 mM Tris–HCl, pH 7.4, 250 mM LiCl, 1% Triton X-100, 0.7% Na-Deoxycholate, 1 mM EDTA) supplemented with protease inhibitor, and twice with TET (0.2% Tween-20 in TE buffer) supplemented with protease inhibitor, and once with TE-NaCl (50 mM NaCl in TE buffer) supplemented with protease inhibitor. Samples were transferred to fresh DNA LoBind tubes after the second to last wash step to reduce background. Beads were resuspended in TT buffer (10 mM Tris, pH 8, 0.05% Tween-20).

#### Library preparation

Strand-specific libraries were prepared using the KAPA hyperprep kit (Roche, cat#7962363001) using half of the reactions from the manufacturer’s instructions up until the ligation step. Chromatin was eluted and reverse cross-linked from Dynabeads by incubating in elution buffer (4% SDS, 0.2 M EDTA, 0.2 M EGTA, 2 M NaCl) supplemented with 20 mg/ml Proteinase K (Thermo Scientific, #catEO0491) and 10 mg/ml RNase (Sigma, cat#R6513). Chromatin was heated for 1 h at 55°C followed by 30 min at 75°C. The elution was transferred to a new DNA LoBind tube and cleaned up with 1:1 Ampure XP beads (Beckman Coulter, cat#A63881) for 15 min at room temperature. Beads were washed twice with 80% EtOH and dried for 1 min, followed by an elution in 0.5× TT. The last HiFi step from the KAPA hyperprep kit was performed according to the manufacturer’s instructions. To perform a double-sided size selection, a 0.56–0.80× Ampure XP bead clean-up was performed. Beads were washed twice with 80% EtOH, followed by elution in 0.5× TT. Concentration and fragment size of the chromatin was confirmed using Qubit (Thermo) and Tapestation (Agilent), respectively. ChIP DNA samples were then sequenced on a NovaSeqXPlus (Illumina).

#### Analysis of ChIP-seq data

All samples were trimmed using Skewer and aligned to the mouse reference genome mm10 using HISAT2. Tag directories were created using the makeTagDirectory command in HOMER, duplicate reads were removed during this step. Nur77 binding peaks were identified by using HOMER peak calling with default settings, Pol II and H3K27ac binding was called using the regions setting (broad peaks). For the peak files, the reproducible peaks in three or two biological replicates were merged using HOMER’s mergePeaks.pl script. The BigWigs were merged using bigwigAverage from deepTools2 [34]. Peaks were annotated to genes using HOMER’s annotatePeaks.pl script using closest gene approach. Motif search analysis on the identified sites was performed using HOMER’s findMotifsGenome.pl script with default setting. In addition, HOMER’s annotatePeaks.pl was used to find specific so-called NurRE and DR5 motifs in the peaks. We included the following custom motif files in the analysis: NurRE motif (TGA-C/T-C/A-TTT-nnnnnn-AAA-G/T-G-T/C-CA) [35] and the DR5 motif (GGTTCAnnnnnAGGTC) [36].

A heatmap visualizing the ChIP-seq signal around Nur77 binding sites was created using deepTools2 [34]. Venn plots were generated with the R package VennDiagram (v1.7.3). Known motif analysis on the identified sites was performed using HOMER’s findMotifsGenome.pl script with default settings. Immediate early genes ChIP-seq datasets used for this analysis were obtained from the GEO database (Atf3; GSM2663861, JunD; GSM2663856, Fos; GSM2663846, cJun; GSM2974660, RXR; GSM2867744, and TRIM33; GSM1067639). Euler plots were generated with the R package eulerr (v7.0.0). Genome browser snapshots were generated using UCSC. The average density plots were made by scaling a group of genes from the transcription start site (TSS) to the terminal exon site (TES) to 5000 bp, with unscaled 1.5 kb/3 kb overhangs with computeMatrix and then plotProfile from deepTools2 [34]. For the Pol II coverage at different regions, we mapped the Pol II ChIP-seq to TSS (−500 bp to +500 bp around TSS), genebody (+500 bp downstream of TSS to +500 bp downstream of the TES), and the TES (+500 bp to +3 kb downstream of the TES) of our gene group of interest. Then the Pol II counts were averaged for those regions and plotted per gene.

To match RNA-seq and ChIP-seq data, we first applied for the ChIP-seq data Homer’s annotatePeaks.pl to assign peaks to the nearest TSS, and thus to the closest Refseq gene. Next, annotatePeaks.pl was used to identify motifs, for example NBRE and AP-1 motifs, in the gene-associated peaks (in a 200-bp region from the peak center). Finally, for those genes that were identified in the RNA-seq experiment to be upregulated by LPS and downregulated by Nur77, we matched their annotated peaks and determined the presence of NBRE/AP-1 motifs in these peaks.

### Rapid immunoprecipitation mass spectrometry of endogenous proteins

#### Sample preparation

The RIME procedure was performed as described previously [37]. In brief, RAW-Ctrl and RAW-HA-Nur77 were seeded in 24 T75 flasks at 3.75 × 10^6^ cells per flask. After 2 days, cells were treated with 50 ng/ml doxycycline for 18 h, followed by a stimulation with 500 ng/ml LPS for 0 and 1 h. Cells were washed twice with PBS, followed by cross-linking with 1% formaldehyde for 10 min at room temperature. Crosslinking reactions were quenched by addition of glycine to a final concentration of 0.13 M and incubated for 2 min at room temperature. Cross-linked cells were washed twice with ice-cold PBS and collected by scraping in ice-cold PBS supplemented with protease inhibitor followed by centrifugation for 10 min at 2000 × *g* and 4°C. A sonication step at +4°C was performed by seven cycles of 30 s on/off in a Biorupter® pico (Diagenode) sonication device. Lysates from four T75 flasks were pooled together, resulting in three biological replicates per condition. Next, the immunoprecipitation was performed as previously described [37]. The nuclear lysates were incubated with 25 µl magnetic protein G Dynabeads (Invitrogen, cat#1003D) conjugated to 7.5 µg of HA antibody (Diagenode, cat#C15200190).

#### Mass spectrometry

For mass spectrometry, peptide mixtures were prepared and measured as previously described [38], with the following exceptions: peptide mixtures (10% of total digest) were loaded directly onto the analytical column and analyzed by nanoLC-MS/MS on an Orbitrap Exploris 480 Mass Spectrometer equipped with a Proxeon nLC1200 system (Thermo Scientific). Solvent A was 0.1% formic acid/water and solvent B was 0.1% formic acid/80% acetonitrile. Peptides were eluted from the analytical column at a constant flow of 250 nl/min in an 80-min gradient, containing a 68-min linear increase from 7% to 26% solvent B, followed by a 12-min wash at 90% solvent B.

#### Analysis of RIME data

Raw data were analyzed by MaxQuant (version 2.2.0.0) [39] using standard settings for label-free quantitation (LFQ). MS/MS data were searched against the Swissprot Mus Musculus database (17 125 entries, release 2022_08) complemented with a list of common contaminants and concatenated with the reversed version of all sequences. The maximum allowed mass tolerance was 4.5 ppm in the main search and 0.5 Da for fragment ion masses. FDR for peptide and protein identification were set to 1%. Trypsin/P was chosen as cleavage specificity allowing two missed cleavages. Carbamidomethylation was set as a fixed modification, while oxidation and deamidation were used as variable modifications. LFQ intensities were log2-transformed in Perseus (version 2.0.7.0) [40], after which proteins were filtered for at least 3 out of 3 valid values in at least one sample group. Missing values were replaced by imputation based on a normal distribution (width: 0.3 and downshift: 1.8). Differentially expressed proteins were determined using a Student’s *t*-test (threshold: FDR: 5% and S0: 0.1). Volcano plots of the identified proteins were created using R studio.

### Assay for transposase-accessible chromatin sequencing

#### ATAC library preparation

ATAC libraries were prepared as previously described with some modifications [41]. In brief, 0.5 × 10^5^ cells were washed in ice-cold 1× PBS and nuclei were isolated using 50 µl of ATAC-RSB (10 mM Tris–HCl, pH 7.4, 10 mM NaCl, 3 mM MgCl_2_) supplied with 0.1% NP-40, 0.1% Tween-20, and 0.01% digitonin. After 3 min the lysis reaction was arrested by adding 1 ml of ATAC-RSB buffer supplemented with 0.1% Tween-20. Nuclei were spun down at 500 × *g* for 10 min at 4°C, and then incubated for 45 min at 37°C under 1000 rpm rotation in 25 µl tagmentation buffer composed of 12.5 µl of 2× TD buffer (20 mM HEPES, pH 7.6, 10 mM MgCl_2_, 20% dimethyl formamide; brought at pH 7.6 with glacial acetic acid), 8.25 µl of 1× PBS, 0.25 µl Tween-20, 0.05 µl of 5% digitonin, 2 µl nuclease-free water, and 1+1 µl of custom Tn5 produced by the NKI in-house Protein Facility. For amplification of the tagmented DNA, two rounds of PCR were performed using the KAPA kit amplification protocol and Nextera dual-indexing sequencing adapters. Fragments <700 bp were purified using SPRI beads (Beckman Coulter) selection. Library quality was assed using the 2100 Bioanalyzer system (Agilent) before sequencing by the NextSeq 500 (Illumina) with 32 × 44 bp paired-end setup.

#### ATAC-seq data analyses

Sequencing reads were de-multiplexed and trimmed using cutadapt (v2.6) [42]. Mapping to mm10 genome and downstream analyses were performed using the snakeATAC (v0.1.1) pipeline available at https://github.com/sebastian-gregoricchio/snakeATAC and using default configuration parameters. Differential binding sites were defined using diffBind (v3.0.15) [43]. Sites with an FDR-adjusted *P*-value <.05 and a log_2_ FC < -1 or > 1 were considered to be differentially regulated. Volcano plots and density plots for the footprinting analysis have been generated using *Rseb* (v.0.3.3) [44]. Known motif analysis on identified sites was performed using HOMER’s findMotifsGenome.pl script with default settings.

### Western blotting

RAW-Ctrl and RAW-Nur77 cells were seeded in six-well plates at 4 × 10^5^ cells per well. The next day, cells were treated with 50 ng/ml doxycycline for 18 h, followed by a stimulation with 500 ng/ml LPS. After stimulation, cells were washed with PBS and lysed in ice cold RIPA buffer (50 mM Tris–HCl, pH 7.4, 150 mM NaCl, 1% IGEPAL CA-630, 0.5% sodiumdeoxycholate, 0.1% SDS) supplemented with protease inhibitor (cOmplete mini EDTA-free; Merck, cat#11836170001) and phosphatase inhibitor (PhosSTOP; Roche, PHOSS-RO). Protein quantification was performed using the DC Protein Assay Kit (Bio-Rad) following the manufacturer’s protocol. Protein samples were heated for 5 min at 96°C. Total protein (15 µg) was separated on a 4%–12% Bolt Bis–Tris gel (Invitrogen, cat#NW04120BOX) and transferred onto a nitrocellulose membrane (Bio-Rad). The membranes were blocked with 2% nonfat milk in 0.1% Tween (v/v) in TBS (TBS-T) for 1 h. The following antibodies were used for western blot staining at a concentration of 1:800: ATF-3 (Santa Cruz, cat#sc-518032) and cFos (Santa Cruz, cat#sc-166940). As a reference protein β-actin (Cell Signaling, cat#4967) was used at 1:3000. Blots were incubated overnight at 4°C, followed by three washes with TBS-T. The protein bands were detected by incubating the membranes with horseradish peroxidase-conjugated secondary antibodies for 1 h at room temperature. Following five washes with TBS-T, the protein bands were visualized using the Supersignal West Pico PLUS chemiluminescent substrate (Thermofisher Scientific, cat#34580) and captured with the ImageQuant 800 western blot imaging system (Amersham).

### Production of zinc-finger mutant plasmids and lentiviral plasmids

The Nur77-ΔZnf1 and Nur77-CE284-285AA (Nur77-CE/AA) variants were generated by site-directed mutagenesis in the pCMV-HA-Nur77 plasmid containing hNur77 complementary DNA (cDNA) (GenBank, D49728, bp 8–1920) following the manufacturer protocol of the AccuPrime *Pfx* DNA Polymerase kit (Invitrogen, cat#12344024). For Nur77-ΔZnf1 the primers (5′-3′) Fw-GAAGTGAAGGCCGCTTCAAGCGCACAGTG and Rv-CACTGTGCGCTTGAAGCGGCCTTCACTTCC were used and for Nur77-CE/AA the primers (5′-3′) Fw-CCAGCATTATGGTGTCCGCACAGCTGCGGGCTGCA AGGG and Rv-CCCTTGCAGCCCGCAGCTGTGCGGAC ACCATAATGCTGG were used. Nur77-ΔZnf1 and Nur77-CE-mutant were cloned into pENTR4-HA plasmid and recombined into pInducer20b following the manufacturer protocol of LR Clonase II (Invitrogen, cat#11791020). All constructs were verified by sequencing.

### Split-luciferase assay

HEK293T cells were seeded in white 96-well plates (Gibco, #65507) at 0.8 × 10^4^ cells per well. The next day, the cells were transfected using Jetprime following manufacturer instructions (Polyplus Sartorius, #101000015) with 50 ng of the following combinations: Nur77-LgBit/HaloTag, Nur77-LgBit/SmBit-RXR, Nur77-LgBit/SmBit-p65, Nur77-LgBit/cJun-SmBit, Nur77-LgBit/cFos-SmBit, SmBit-cFos/cJun-LgBit, and SmBit-p65/LgBit-p65. After 48 h, NanoBiT PPI Nano-Glo® Live Cell Reagent, a non-lytic detection reagent containing the cell-permeable furimazine substrate, (Promega, cat#N2011) was used to measure protein–protein interaction in intact cells.

### Luciferase assay

HEK293T cells were seeded at a density of 4 × 10^4^ cells per well in 24-well plates. After culturing for 18–20 h, cells were transfected with either an NBRE-reporter (400 ng) or an AP-1 reporter plasmid (200 ng) using the JetPrime transfection reagent kit (PolyPlus Sartorius, #101000015). For NBRE luciferase assays, cells were additionally transfected with 20 ng of pCMV-Nur77, pCMV-Nur77-ΔZnf1, or pCMV-Nur77-CE-mutant. For AP-1 luciferase assays, cells were co-transfected with 50 ng of cJun-SmBit or cFos-SmBit and 50 ng of pCMV-Nur77, pCMV-Nur77-ΔZnf1, or pCMV-Nur77-CE-mutant. In all experiments, Tk-Renilla was co-transfected to correct for transfection efficiency, and pCMV-Myc empty vector was used as a negative control. Forty-eight hours post-transfection, cells were lysed with Passive Lysis Buffer according to the Dual-Luciferase Reporter Kit instructions (Promega, cat#E1910). Following lysis, 10 µl of cell lysate was transferred to a white flat-bottom 96-well plate. Luciferase activity was measured using the GloMax Multi Detection System (Promega, #9301-062) according to the Dual Luciferase Reporter Kit instructions. Results were normalized for transfection efficiency by dividing Firefly luciferase values by the respective *Renilla* luciferase values.

### Reverse transcription-qPCR

For BMDMs and RAW264.7 cells, total RNA was extracted from stimulated cells using TRIreagent (Sigma), adhering to the manufacturer’s protocol. The resulting RNA pellet was reconstituted in nuclease-free ultrapure water. RNA concentrations were measured with a NanoDrop UV-VIS Spectrophotometer. For reverse transcription (RT), 1 µg of RNA was converted to cDNA using the iScript cDNA Synthesis Kit (Bio-Rad). Quantitative PCR (qPCR) was conducted with the SensiFAST SYBR No-ROX kit (Bioline) on a LightCycler 480 II PCR platform (Roche). Primer set amplification efficiencies and initial transcript concentrations (N0) were determined using LinRegPCR software [45]. Expression levels of target genes were normalized to the geometric mean of *Rplp0* and *Hrpt* housekeeping genes. The primer sequences used for RT-qPCR are *IL-1β*-CAGGCAGGCAGTATCACTCA/AGCTCATATGGGTCCGACAG, *Il6*-CCGGAGAGGAGACTTCACAG/CAGAATTGCCATTGCACAAC, *Il12b*-AGCACGGCAGCAGAATAAAT/TTTCTTTCTTGCGCTGGATT, *Rplp0*-GGACCCGAGAAGACCTCCTT/GCACATCACTCAGAA TTTCAATGG, and *Hprt*-TTGCTCGAGATGTCATGAAGGA/AGCAGGTCAGCAAAGAACTTATAG.

### Quantification and statistical analysis

For the cytokine assay, Pol II coverage, and western blot FC calculation, a one-way ANOVA followed by Tukey multiple comparisons test was performed (GraphPad Prism 10.2.0). For LPS fold enrichment of Pol II, H3K27ac, and ATAC-seq, a Mann–Whitney *U* test was used (GraphPad Prism 10.2.0). For the luciferase and qPCR data, an unpaired *t*-test was performed (GraphPad Prism 10.2.0). All bar graphs show mean ± SD (error bars). All box plots indicate the median (center line), upper (75), and lower (25) quartile range (box limits), and a minimum/maximum range (whiskers). Significance is indicated as follows: ns non-significant; *P < .05, **P < .01, ***P < .001, ****P < .0001.

## Results

### Nur77 inhibits the expression of targets of AP-1 transcription factors during macrophage activation

**Figure 1.**
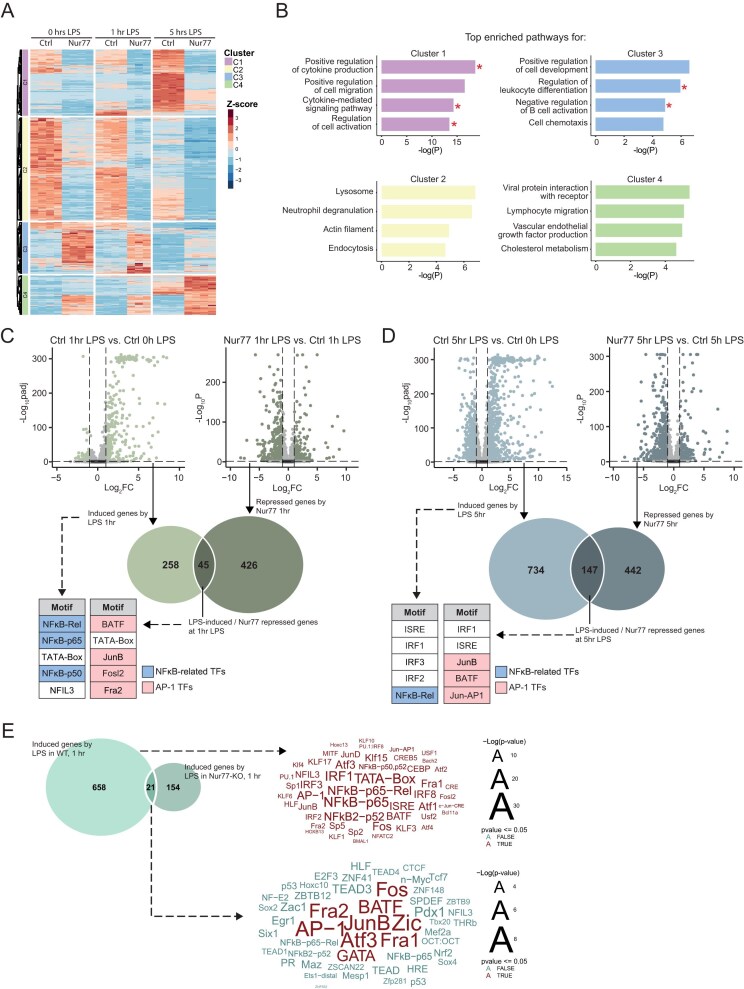
Nur77 inhibits LPS-induced activation of macrophages in RNA-seq analyses. (**A**) Hierarchical clustering of genes and *z*-score heatmaps of differentially expressed genes between RAW-Ctrl and RAW-Nur77 cells at 0, 1, and 5 h of LPS stimulation as determined by RNA-seq. Color annotation represents the unbiased clustering of four groups [Cluster (C) 1–4]. Color scale indicates gene expression (*z*-score). (**B**) Top four enriched pathways in RAW-Nur77 compared to RAW-Ctrl for genes of the four clusters defined in panel (A). Pathway analysis was performed in Metascape. Red asterisks represent pathways involved in inflammatory processes. (**C**) Scatterplots of RNA-seq data showing 1 h LPS-regulated gene expression in RAW-Ctrl cells (left panel, light green) and Nur77-regulated gene expression by comparing RAW-Nur77 cells with RAW-Ctrl cells at 1 h of LPS stimulation (right panel, medium green). The overlap between genes significantly induced by LPS in RAW-Ctrl cells (*n* = 303, *P*-adj <.05; log_2_ FC > 1) and Nur77-repressed genes in RAW-Nur77 cells compared to RAW-Ctrl after 1 h of LPS stimulation (*n* = 471, *P*-adj < .05; log_2_ FC < -1) is shown by Venn Diagrams. The group of overlapping genes that are LPS-induced/Nur77-repressed is shown in dark green (*n* = 45). Known-motif analysis of LPS-induced genes in RAW-Ctrl cells (*n* = 303) and LPS-induced/Nur77-repressed genes (*n* = 45) is shown in Tables. Significance cutoffs are shown as dotted lines. (**D**) Scatterplots of RNA-seq data showing 5 h LPS-regulated gene expression in RAW-Ctrl cells (left panel, light blue) and Nur77-regulated gene expression by comparing RAW-Nur77 cells with RAW-Ctrl cells at 5 h of LPS stimulation (right panel, medium blue). The overlap between significant LPS-induced genes in RAW-Ctrl cells (*n* = 881, *P*-adj <.05; log_2_ FC > 1) and Nur77-repressed genes in RAW-Nur77 cells compared to RAW-Ctrl after 1 h of LPS stimulation (*n* = 589, *P*-adj <.05; log_2_ FC < -1) is shown by Venn Diagrams. The group of shared genes indicated as LPS-induced/Nur77-repressed is shown in dark blue (*n* = 147). Known motif analysis of LPS-induced genes in RAW-Ctrl cells (*n* = 881) and LPS-induced/Nur77-repressed genes is shown in Tables. Significance cutoffs are shown as dotted lines. (**E**) Venn-diagram depicting the overlap between LPS-induced genes in WT BMDMs (*n* = 679, *P*-adj <.05; log_2_ FC > 1) and genes induced in Nur77-deficient BMDMs after 1 h of LPS stimulation (*n* = 175, *P*-adj <.05; log_2_ FC > 0.58). Word clouds showing known-motif enrichment analysis in the promoters of 1 h LPS-induced genes in WT BMDMs and in the promoters of genes induced by LPS even more (>1.5-fold) in BMDM-Nur77-KO cells. Font sizes represent the −log(*P*-value) and colors correspond to significance (dark red = *P*-value ≤.05, blue = *P*-value >.05).

It is well-established that Nur77 deficiency in macrophages promotes a pro-inflammatory phenotype when these cells are activated [20, 26, 46]. To delineate the underlying mechanism of Nur77-mediated inhibition of the inflammatory response of macrophages, we performed extensive studies in RAW264.7 macrophages. We previously generated RAW264.7 cell lines with inducible overexpression of either hemagglutinin (HA)-tagged Nur77 (RAW-Nur77) or GFP as a control (RAW-Ctrl) [26] (Supplementary Fig. S1A). In this prior investigation on the role of Nur77 in RAW264.7 macrophages, the cells were analyzed under non-stimulated conditions, whereas in the current study, the impact of Nur77 on activated macrophages was investigated. To validate the anti-inflammatory effect of Nur77 overexpression on activation of these macrophages, we analyzed the secretion of various cytokines in the cell supernatant. RAW macrophages were either left untreated or stimulated with LPS for 18 h. As expected, Nur77 overexpression reduced secretion of a number of cytokines and chemokines crucial in the inflammatory response of macrophages: IL-6, IL-1β, IL-12p70, IL-23, IL-17, and IFN-γ (Supplementary Fig. S1B). To unravel the molecular mechanism of Nur77 action in activated macrophages, we analyzed RNA expression dynamics in RAW-Ctrl and RAW-Nur77 macrophages in response to 1 and 5 h of LPS treatment [principal component analysis (PCA) in Supplementary Fig. S1C]. Unbiased hierarchical clustering of the RNA-seq data resulted in four distinct subsets of genes (Fig. 1A). Genes in cluster 1 (C1), are induced by LPS stimulation in the RAW-Ctrl cells, whereas Nur77 overexpression in turn robustly reduces the expression level of these transcripts (Fig. 1A). Pathway analysis of the genes in C1 reveals the association with downregulation of inflammatory pathways, representing the Nur77-mediated repression of LPS-induced inflammation (Fig. 1B). The genes in cluster 2 (C2) are downregulated by Nur77 under all three conditions: 0, 1, and 5 h of LPS stimulation. Of interest, pathway analysis for these genes highlights their association with cellular mechanics, a process that we showed to be affected by Nur77 (Fig. 1B) [47]. Genes represented in clusters 3 and 4 (C3, C4) are found to be increased in Nur77 overexpressing macrophages compared to the control cells.

To gain further insight into the gene regulation by LPS and/or Nur77, we identified genes in RAW-Ctrl cells that are induced by LPS and reduced upon overexpression of Nur77. Of the 303 mRNA transcripts that are induced >2-fold upon 1 h of LPS exposure (FDR < 0.05) in Ctrl cells, the expression of 45 genes (14.9%) was downregulated by Nur77 (Fig. 1C). After 5 h of LPS stimulation, 147 of the 881 LPS-induced mRNA transcripts (16.7%) were reduced upon overexpression of Nur77 (Fig. 1D and Supplementary Table S1). The expression of only 5.2% (at 1 h) and 4.0% (at 5 h) of the genes induced by LPS was further increased in response to Nur77 (Supplementary Fig. S1D and E). This indicates that Nur77 mostly has a repressive effect on LPS-induced genes. Next, we performed a motif analysis of the promoter regions of genes induced by LPS, revealing the presence of especially NF-kB motifs at 1 h and motifs that match the secondary interferon response [interferon regulatory factors (IRFs)] at 5 h LPS (Fig. 1C and D; and Supplementary Fig. S1F and G). Unexpectedly, no NF-kB-motif was found in promoters of the LPS-induced/Nur77-repressed genes at both time points (Fig. 1C and D; and Supplementary Fig. S1H and I). This is remarkable because it has been proposed that the mechanism of Nur77-mediated inhibition of inflammation is the result of NF-kB transrepression [46, 48]. In the current analyses, Nur77 overexpression appears to especially inhibit the expression of LPS-induced genes containing AP-1 motifs (1 and 5 h) and IRF motifs (5 h) in their promoter region (Fig. 1C and D; and Supplementary Fig. S1H and I). To further substantiate our observations and overcoming the disadvantage of studying macrophages overexpressing Nur77, we extended our analyses to BMDMs from wild-type (BMDM-WT) and Nur77-deficient (BMDM-Nur77-KO) mice. BMDM-WT and BMDM-Nur77-KO were treated with LPS for 1, 5, and 18 h (PCA analysis in Supplementary Fig. S1J). Gene set enrichment analyses (GSEA) for genes regulated in Nur77-deficient BMDMs compared to WT BMDMs revealed increased expression of genes in pro-inflammatory pathways (Supplementary Fig. S1K), in line with the observation that overexpression of this nuclear receptor reduces inflammatory pathways. Unbiased hierarchical clustering of BMDM RNA-seq data in eight distinct subsets of genes and subsequent pathway analyses of these clusters confirms in further detail the pro-inflammatory status of BMDMs upon Nur77 deficiency (Supplementary Fig. S1L and M). Next, we performed motif analyses in the promoter regions of 658 genes that are induced by LPS after 1 h of stimulation in WT BMDMs. This clearly shows enrichment of both NF-kB and AP-1 motifs, as may be expected (Fig. 1E). In Nur77-deficient BMDMs, we observed an enrichment of AP-1 motifs at promoters of LPS-upregulated genes, which were even stronger upregulated in Nur77-deficient cells (Fig. 1E). Comparable analyses were performed at the other time points for BMDMs focusing on LPS-induced genes that are further induced upon Nur77-deficiency (Fig. Nur77-deficiencyN). These data are in agreement with our observation in Nur77-overexpressing macrophages with enrichment of AP-1 motifs at promoters of LPS-induced/Nur77-*repressed* genes.

Next, to combine the transcriptomic information derived from macrophages overexpressing Nur77 and Nur77-deficient macrophages, we searched for LPS-induced/Nur77-repressed genes in RAW264.7 cells that also exhibit increased expression in Nur77-deficient BMDMs treated with LPS compared to WT BMDMs treated with LPS. Even though the overexpression experiment was performed in the stable, macrophage cell line RAW264.7 cells and primary BMDMs were used to assess the impact of Nur77 deficiency, this analysis revealed 66 genes showing an opposite expression pattern. These genes are highlighted in Supplementary Table S1. The typical expression pattern of these genes is illustrated in heatmaps (Supplementary Fig. S1O). GSEA of this set of genes revealed their connection to the adaptive immune system supporting the anti-inflammatory function of Nur77 in macrophages (Supplementary Fig. S1P).

Taken together, we confirm that Nur77 overexpression inhibits the inflammatory response of activated macrophages, while Nur77 deficiency enhances the inflammatory response. Our RNA-seq experiments identified a specific cluster of LPS target genes that is downregulated by Nur77 and showing enhanced expression upon Nur77 deficiency. Promoter motif enrichment analyses suggest that Nur77-mediated inhibition of these genes is independent of NF-kB but involves immediate-early genes like AP-1 and IRF family members. Importantly, these results were confirmed in Nur77-deficient macrophages, where AP-1 motifs are enriched at promoters of LPS-induced genes with even higher expression upon Nur77 deficiency.

### Nur77 recruitment to chromatin changes after LPS stimulation and Nur77 colocalizes with AP-1 factors

**Figure 2.**
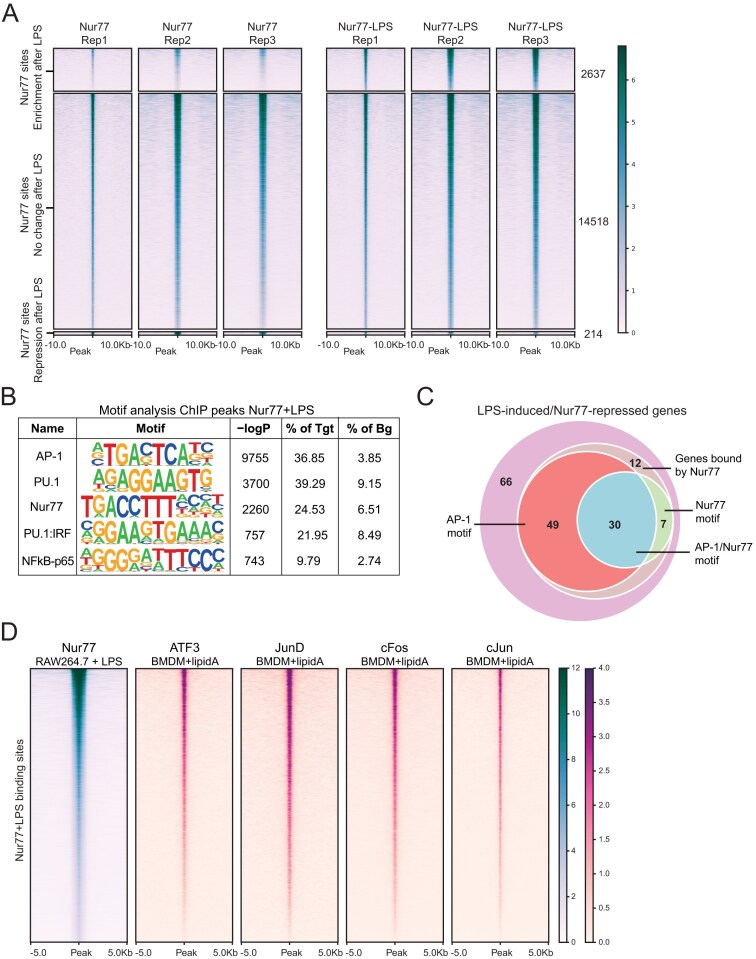
Genome-wide chromatin recruitment of Nur77 to chromatin shows co-localization with AP-1 motifs. (**A**) Tornado plots visualize Nur77 ChIP-seq signal for the three replicates before and after LPS in a 20-kB window around the peak center. The binding sites were divided into three groups: (i) Enriched binding of Nur77 after LPS stimulation (Nur77+LPS versus Nur77, *P*-value <.05; FC > 2), (ii) no change in binding after LPS, and (iii) loss of binding after LPS (Nur77+LPS versus Nur77, *P*-value <.05; FC < 0.5). The replicates were merged into Nur77 binding sites before (*n* = 13 596) and after LPS stimulation (*n* = 17 370). (**B**) Top-enriched known transcription factor motifs within 200 bp of Nur77 peaks after LPS stimulation. (**C**) Euler diagram showing genes from the LPS-induced/Nur77-repressed group at both 1 and 5 h of LPS stimulation as determined by RNA-seq (*n* = 164) that contain a Nur77 binding site (*n* = 98). The LPS-induced/Nur77-repressed genes have Nur77 binding at an AP-1 motif, a Nur77 motif, or at both motifs, or at none of these motifs. (**D**) Tornado plots showing Nur77+LPS ChIP-seq signal (*n* = 17 370) together with publicly available ChIP-seq data for ATF3, JunD, cFos, and cJun in BMDMs after 1 h of lipidA stimulation.

To define the genome-wide landscape of Nur77 recruitment to chromatin before and after 1 h of LPS stimulation and assess the motifs bound by HA-tagged overexpressed Nur77, we performed ChIP-seq (Fig. 2A and Supplementary Fig. S2A). For these experiments we used an antibody directed against the HA-epitope tag as no Nur77-specific antibodies are currently available. In total, 2637 (15.2%) of total Nur77 sites were enriched, whereas only 214 sites (1.2%) were reduced after LPS stimulation (*P*-value <.05, FC > 2). This dynamic recruitment of Nur77 to chromatin may be crucial to execute its anti-inflammatory function in stimulated macrophages. Motif analysis of Nur77 binding sites after LPS stimulation revealed highly significant enrichment of the AP-1 motif, exceeding that of the canonical monomeric NBRE Nur77 motif itself (Fig. 2B). This observation aligns with Nur77’s association with targets of these transcription factors in the RNA-seq data (Fig. 1C and D). When studying motifs enriched at Nur77 binding sites after LPS stimulation, there are significantly more AP-1 motifs found than before LPS stimulation (Supplementary Fig. S2B). To assess whether Nur77 is recruited to the chromatin near LPS-induced/Nur77-repressed genes (Fig. 1C and D), we overlaid Nur77 ChIP-seq and RNA-seq data. This revealed that for more than half of the LPS-induced/Nur77-repressed genes identified at 1 and 5 h of LPS stimulation identified by RNA-seq, indeed Nur77 peaks were annotated in our ChIP-seq; 98 out of 164 genes (59.8%; Fig. 2C). Next, we asked whether these Nur77 peaks contain Nur77 or AP-1 motifs. Interestingly, more peaks contain AP-1 motifs (49 out of 98 genes; 50.0%) than Nur77 motifs (7 out of 98 genes; 7.1%), suggesting that Nur77 often interacts with chromatin via an indirect mode. Of note, in some cases multiple Nur77 peaks were annotated to the same gene, with either multiple AP-1 or Nur77 motifs, or both motifs (*n* = 30). To gain more insight to which loci Nur77 is recruited near LPS-induced/Nur77-repressed genes, we determined the genomic distribution of Nur77 binding (Supplementary Fig. S2C). Nur77 peaks after LPS stimulation are primarily located at gene bodies (46%), intergenic regions (41%), and promoter regions (12%). Nur77 has been described to also bind motifs that are not represented in the commonly used HOMER datasets; the so-called NurRE motif binding Nur77 dimers [35] and the DR5 motif allowing interaction of Nur77 as a heterodimer with RXR [36]. We analyzed our HA-Nur77 ChIP-seq data for these specific motifs (see the “Materials and methods” section for details). The NBRE motif was detected in 4717 and 3360 Nur77-binding sites before and after LPS stimulation, respectively. These numbers are substantially lower for the NurRE motif (264 and 206, respectively) and for the DR5 motif (143 and 110, respectively) (Supplementary Fig. S2D). Genes identified by RNA-seq as LPS-induced/Nur77-repressed and presented in Fig. 2C with HA-ChIP-seq data were also analyzed for NurRE and DR5 motifs with six and one hits, respectively (Supplementary Table S2). Intriguingly, the Nur77 homodimer or Nur77/RXR-heterodimer motifs (NurRE/DR5) seem underrepresented compared to the NBRE-motif. This may be related to the fact that the search criteria we applied for the NurRE/DR5 motifs were very stringent, whereas these palindromic sites often have one half-site better conserved than the other without diminishing their functionality. Most likely, DR5/NurRE motifs are actually present at sites that were labeled as “NBRE.” So far, the relative contribution of Nur77 dimerization in its function is unknown and challenging due to the mere fact that no “monomer-only variant” of Nur77 has been identified.

To further explore whether the Nur77 peaks are enriched for AP-1 motifs, we performed analyses incorporating publicly available ChIP-seq data for the AP-1 family members ATF3, JunD, cFos, and cJun in macrophages, activated with LPS-component lipidA [49]. We found a remarkable overlap in loci at the chromatin recruiting Nur77 and these AP-1 members (Fig. 2D).

In conclusion, in activated macrophages the cistromes of Nur77 and AP-1 factors show remarkable overlap at enhancers and to a lesser extent at promoters. As the majority of these sites do not contain any of the Nur77-binding motifs, this raises the question whether there are other proteins or co-regulators needed for Nur77 to perform its anti-inflammatory function.

### Analysis of the Nur77 interactome

**Figure 3.**
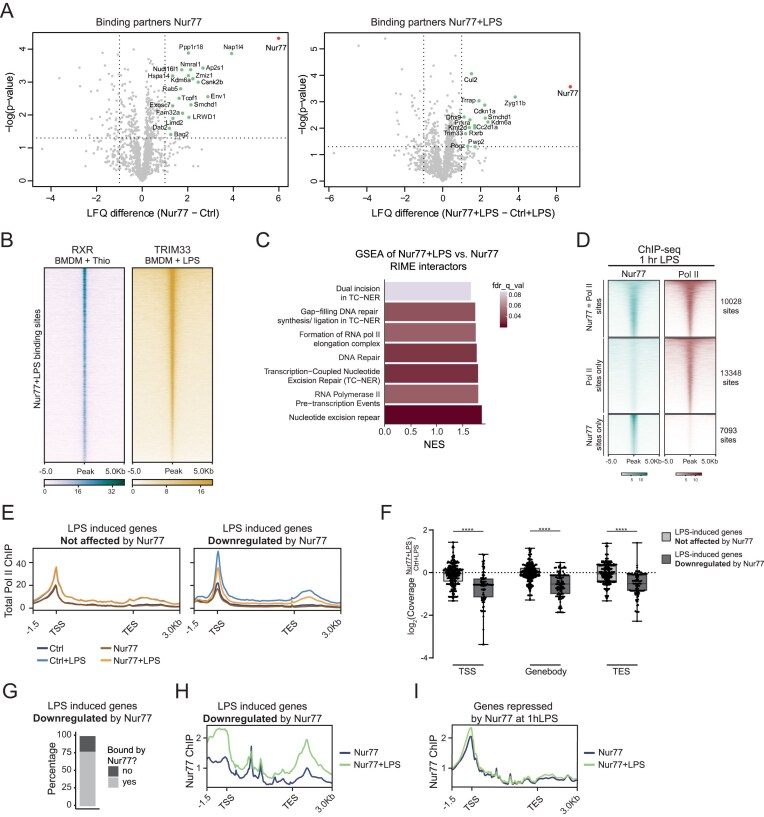
Nur77 protein associates with RNA polymerase II (Pol II) regulatory complexes and limits Pol II binding at LPS-induced/Nur77-repressed genes. (**A**) Volcano plots showing HA-Nur77 protein–protein interactions as determined by RIME before and after LPS stimulation in RAW-Nur77 cells over RAW-Ctrl cells. Significantly enriched proteins interacting with HA-Nur77 are highlighted and labeled. Significance cutoffs are shown as dotted lines (LFQ difference ≥ 1; *P* ≤ .05, *n* = 3). (**B**) Tornado plots showing the alignment of publicly available ChIP-seq data for RXR in thioglycolate-elicited peritoneal macrophages and TRIM33 in LPS-stimulated BMDMs at Nur77+LPS binding sites. (**C**) GSEA pathway analysis for Nur77-interacting proteins enriched post-LPS stimulation compared to pre-LPS stimulation. The *X*-axis indicates the normalized enrichment score (NES) and bar colors indicate the FDR values. (**D**) Alignment of ChIP-seq data for Nur77 and Pol II reveals shared sites (*n* = 10 028), Pol II unique sites (*n* = 13 348), and sites that are unique for Nur77 (*n* = 7093) in RAW-Nur77 cells after 1 h of LPS stimulation. Data are centered around peaks in a 10-kb window. (**E**) Average density plots depicting Pol II ChIP-seq signal at LPS-induced genes not affected by Nur77 overexpression or LPS-induced/Nur77-repressed genes (defined in our RNA-seq). Lines shown represent the Pol II signals from RAW-Ctrl (dark blue), RAW-Ctrl+LPS (light blue), RAW-Nur77 (dark orange), or RAW-Nur77+LPS (light orange) cells over a scaled distance between the TSS and TES with 1.5 kb/3 kb overhangs. (**F**) Box plots showing the ratio of ChIP-seq signal coverage of Pol II in RAW-Nur77 over RAW-Ctrl cells after 1 h of LPS stimulation at the TSS, gene body, and TES for LPS-induced genes not affected by Nur77 or for the LPS-induced/Nur77-repressed genes. ****P < .0001 (One-way ANOVA with Tukey’s post-hoc test). (**G**) Stacked bar chart depicting the percentage of LPS-induced/Nur77-repressed genes bound by Nur77 anywhere in or near the gene. (**H**) Average density plot depicting Nur77 HA-ChIP-seq signal at LPS-induced/Nur77-repressed genes. The lines shown represent the Nur77 signal from RAW-Nur77 (blue) or RAW-Nur77+LPS (green) over a scaled distance between the TSS and TES with 1.5 kb/3 kb overhangs. (**I**) Average density plot depicting Nur77 HA-ChIP-seq signal at Nur77 repressed genes (versus Ctrl) at 1 h LPS-treatment. The lines shown represent the Nur77 signal from RAW-Nur77 (blue) or RAW-Nur77+LPS (green) over a scaled distance between the TSS and TES with 1.5 kb/3 kb overhangs.

Next, we investigated the Nur77 interactome to identify binding partners that may be critical in shaping the Nur77-mediated, less inflammatory macrophage state. To identify Nur77 binding partners, we employed a RIME experiment [37]. Specifically, RIME assays were conducted on RAW-Nur77 cells before and after 1 h of LPS stimulation, utilizing RAW-Ctrl cells as background controls (Supplementary Fig. S3A). We identified endogenous binding partners of overexpressed Nur77 in the absence of LPS and after LPS stimulation (Fig. 3A; left and right panel, respectively). A complete list of the identified binding partners that were specifically enriched before and after LPS stimulation is provided (Supplementary Fig. S3B). As expected, Nur77 was detected with high significance in both experiments. In addition, we identified retinoid receptor X (RXR), a known, validated binding partner of Nur77, which was pulled down in LPS-stimulated cells [36, 50] (Fig. 3A; right panel). Based on the DNA-binding profile of Nur77 at AP-1 motifs and its chromatin colocalization with such transcription factors, we hypothesized that Nur77 may interact with these AP-1 factors on DNA. Unexpectedly, no members of the AP-1 family were pulled down with significant difference. The only AP-1 factor that was detected is cJun, showing some interaction with Nur77 after LPS stimulation with a non-significant 1.8-fold increase (Nur77-LPS/GFP-LPS, *P*-value 0.57). No NF-kB subunits were identified in the RIME data, even though Nur77-mediated inhibition of inflammation has been attributed to its interaction with NF-kB transcription factor RelA/p65 [46–48, 51]. However, several transcription factor co-regulators were identified in the Nur77 protein complex, such as the E3 ligase Trim33 that stabilizes estrogen receptor alpha (ERα) and the androgen receptor (AR) [52, 53]; the lysine demethylases Kdm6a and Kdm6b; the H3K4-demethylase Kmt2d; and co-repressor Smchd [54]. Especially Kdm6a is of interest, as microglia-selective deficiency of this X-linked gene in female mice gave protection against multiple sclerosis, suggesting a pro-inflammatory function of Kdm6a in these cells [55]. To validate our RIME data, we overlaid publicly available ChIP-seq data for identified interactors Trim33 and RXR in macrophages, with our HA-Nur77 ChIP-seq data (Fig. 3B). The analysis revealed substantial genome-wide overlap between Nur77 and these proteins, indicating potential cooperative recruitment of these proteins with Nur77 on chromatin, as detected in the RIME experiment.

To further delineate the Nur77 interactome changes after LPS stimulation, a pathway analysis was conducted for binding partners post-LPS compared to the pre-stimulation state. This analysis revealed the enrichment of multiple pathways associated with RNA polymerase II (Pol II), including the formation of “Pol II elongation complex” and “Pol II Pre-transcription events” (Fig. 3C). Furthermore, pathways linked with nucleotide excision repair, a process known to intersect with Pol II elongation [56], were enriched. Previously, Guo *et al*. indicated Nur77’s association with Pol II in cancer cells [57]. More specifically, it was shown that Nur77 localizes to the gene body of a number of genes causing Pol II stalling, thereby halting transcription of these genes. In response to acute replication stress, Nur77 was shown to dissociate from the genes allowing instantaneous release of halted pre-RNAs leading to immediate expression of these genes. To gain a deeper understanding of Nur77-mediated gene regulation in macrophages, we decided to further study whether Nur77 regulates Pol II in a similar way in these cells. Therefore, we performed a ChIP-seq for Pol II in unstimulated and LPS-stimulated RAW-Ctrl or RAW-Nur77 cells (Supplementary Fig. S3C). The overlay of ChIP-seq data for Nur77 and Pol II resulted in the identification of 10 028 sites that are shared by Nur77 and Pol II, 13 348 sites unique for Pol II, and another 7093 regions only occupied by Nur77 (Fig. 3D). To assess whether Nur77 affects the binding of Pol II to DNA, we outlined the Pol II ChIP-seq data over two groups of genes identified in the RNA-seq experiment. First, the profile of Pol II ChIP-seq was plotted over LPS-induced genes unaffected by Nur77 (Fig. 3E; left panel). The Pol II signal is higher after LPS stimulation, especially around the TSS, which is similar for conditions with and without Nur77. However, genes that are LPS-induced/Nur77-repressed show a lower Pol II signal upon Nur77 overexpression, particularly post-stimulation, indicating reduced Pol II recruitment to these genes (Fig. 3E; right panel). Upon quantification, Pol II binding is indeed lowered by overexpression of Nur77 at the TSS, the gene body, and at the TES of this group of genes compared to genes of which their expression is not affected by Nur77 (Fig. 3F). Based on these data, we concluded that Nur77-mediated inhibition of pro-inflammatory genes in macrophages does not involve Pol II halting as shown in metastatic cancer cells [57], but rather limiting Pol II binding. Next, we established that based on our ChIP-seq data, Nur77 is present at or near ~75% of the genes belonging to the group of LPS-induced/Nur77-repressed genes with a reduced Pol II profile (Fig. 3G). Profiling Nur77 binding in the same manner as Pol II binding showed increased Nur77 binding post-LPS stimulation in RAW-Nur77 cells with a very similar profile as Pol II, with the highest binding around the TSS and TES of genes (Fig. 3H). As a control, we profiled Nur77 binding over all genes that were identified in our RNA-seq as Nur77 repressed genes (versus Ctrl) at 1 h LPS-treatment (Fig. 1C). For these genes we again observed a similar profile as for Pol II in panel (E), however, there is no difference in Nur77 binding before and after LPS treatment (Fig. 3I).

Together, these data indicate that Nur77 seems to repress the expression of LPS-induced/Nur77-repressed genes in macrophages by reducing Pol II occupancy to these genes.

### Nur77-mediated inhibition of expression of AP-1 target genes entails reduced chromatin accessibility

**Figure 4.**
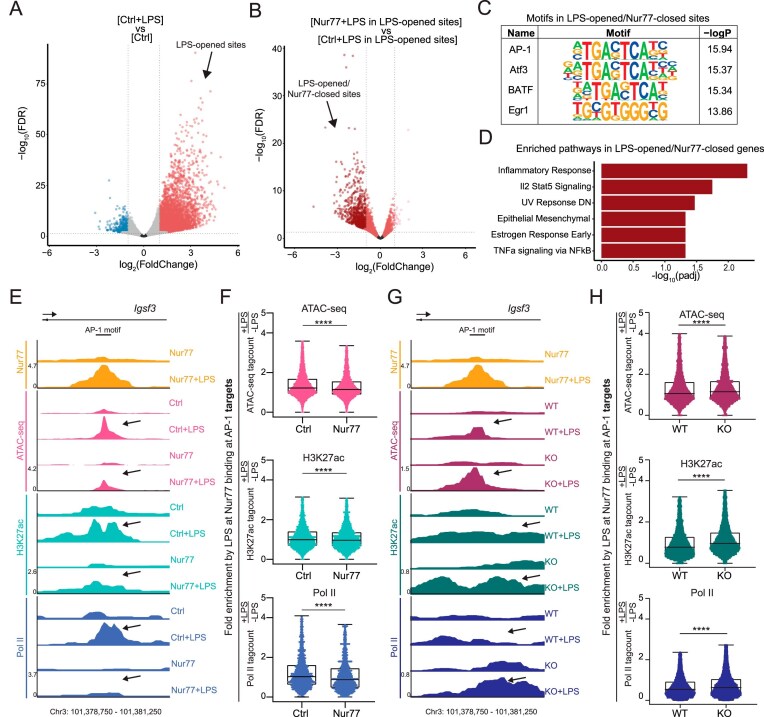
Nur77 overexpression/deficiency in macrophages regulates expression of AP-1 targets by modulating chromatin accessibility, Pol II coverage, and H3K27 acetylation. (**A**) Volcano plot showing the accessibility of chromatin sites in LPS-stimulated RAW-Ctrl cells over RAW-Ctrl cells as determined by ATAC-seq. Significance cutoffs are shown as dotted lines (FC ≥ 2; *P* ≤ .05, *n* = 3). Sites with enriched chromatin accessibility in LPS-stimulated RAW-Ctrl cells are shown in red; these are so called LPS-opened sites. (**B**) Volcano plot showing the accessibility of chromatin sites in LPS-stimulated RAW-Nur77 cells over LPS-stimulated RAW-Ctrl cells. Significance cutoffs are shown as dotted lines (FC ≥ 2; *P* ≤ .05, *n* = 3). Sites with suppressed chromatin accessibility in LPS-stimulated RAW-Nur77 cells compared to LPS-stimulated RAW-Ctrl cells are shown in dark red and are referred to as LPS-opened/Nur77-closed sites. (**C**) Top enriched known transcription factor motifs within 200 bp of LPS-opened/Nur77-closed ATAC-seq sites. (**D**) Bar plot showing top differentially enriched pathways in genes that contain LPS-opened/Nur77-repressed sites using gene ontology (GO) analysis for HALLMARK gene sets. *Y*-axis indicates the *P*-adj. (**E**) Representative example snapshots of Nur77 ChIP-seq (yellow), ATAC-seq (pink), H3K27ac ChIP-seq (turquoise), and Pol II ChIP-seq (purple) at the beginning of the *Igsf3* gene. The *Y*-axis indicates the sequencing signal in fragments per kb per million reads mapped. (**F**) For Nur77 peaks at AP-1 motifs the tag counts of ATAC-seq, H3K27ac, or Pol II ChIP-seq were determined and a ratio was calculated for RAW-Ctrl+LPS over RAW-Ctrl or RAW-Nur77+LPS over RAW-Nur77 and represented in box plots. ****P < .0001 (Mann–Whitney test). (**G**) Representative example snapshots of Nur77 ChIP-seq (yellow) as determined in RAW-Nur77 cells at the beginning of the *Igsf3* gene. For WT and Nur77-KO BMDMs, ATAC-seq (dark pink), H3K27ac ChIP-seq (dark turquoise), and Pol II ChIP-seq (dark purple) are shown at the same gene location. The *Y*-axis indicates the normalized sequencing signal in fragments per kb per million reads mapped. (**H**) For genome-wide Nur77 peaks at AP-1 motif (as determined in RAW-Nur77 HA-ChIP-seq) the tag counts of ATAC-seq, H3K27ac, or Pol II ChIP-seq were determined and a ratio was calculated for BMDM-WT+LPS over BMDM-WT control (WT) and for BMDM Nur77-KO+LPS over BMDM-Nur77-KO control (KO) and represented in box plots. ****P < .0001 (Mann–Whitney test).

To further analyze in an unbiased way changes in chromatin conformation and identify additional transcription factors involved in Nur77-mediated inhibition of gene expression, we sought to uncover whether chromatin accessibility is affected. To explore this, ATAC-seq experiments were conducted on RAW-Ctrl and RAW-Nur77 cells before and after 1 h of LPS stimulation (Supplementary Fig. S4A). As expected, a striking increase in open-chromatin sites was observed in RAW-Ctrl cells in response to LPS and we depicted these sites as “LPS-opened sites” (Fig. 4A, highlighted in red). Next, we determined the chromatin status of these LPS-opened sites upon Nur77 overexpression and observed that a substantial number of these chromatin sites became less accessible (Fig. 4B; highlighted in dark red). Subsequent motif analysis of these so-called “LPS-opened/Nur77-closed sites” revealed a prevalent presence of AP-1 and Egr1 motifs (Fig. 4C and Supplementary Fig. S4B). Pathway analysis for the genes associated with LPS-opened/Nur77-closed chromatin confirmed their association with inflammatory pathways, as expected from targets of these immediate-early transcription factors in macrophages (Fig. 4D). As it appears that Nur77 affects the expression of AP-1 targets, we illustrated this using our ChIP-seq and ATAC-seq data and one representative site is shown as typical example: the Immunoglobulin superfamily member 3 (*Igsf3*) locus (Fig. 4E). Nur77 is present at an AP-1 motif in the promoter region in LPS-activated macrophages. In response to LPS, the chromatin becomes more accessible, whereas Nur77 overexpression results in local loss of chromatin accessibility as seen in the ATAC-seq data. To further study the chromatin environment and identify transcriptionally active genes, we performed ChIP-seq for the histone modification H3K27 acetylation (H3K27ac), which demarcates active promoters and enhancers (Supplementary Fig. S4C). H3K27ac ChIP-seq data follow a pattern similar to the ATAC-seq data, in which the H3K27ac signal is induced upon LPS stimulation, followed by a reduction with Nur77 overexpression. This is indicative of active, local chromatin remodeling by Nur77. In concordance with this pattern, the Pol II coverage of the *Igsf3* locus is increased after LPS stimulation, with reduced Pol II binding in response to Nur77 overexpression (Fig. 4E). To study these effects in a genome-wide manner, we quantified the enrichment of ATAC-seq, Pol II coverage, and H3K27ac coverage at all genes that are bound by Nur77 at an AP-1 motif (defined as AP-1 targets). This analysis demonstrates genome-wide that when Nur77 covers an AP-1 site in LPS-stimulated macrophages, this results in reduced chromatin accessibility, reduction of Pol II binding, and lowered H3K27 acetylation (Fig. 4F). Next, to validate these findings under more physiological conditions, we performed ATAC-seq, H3K27ac ChIP-seq, and Pol II ChIP-seq analyses in WT and Nur77-deficient BMDMs before and after LPS stimulation (Supplementary Fig. S4D and E). At the *Igsf3* locus where an AP-1 motif is present in a Nur77 peak (in yellow), we observed that chromatin accessibility, H3K27ac signal, and Pol II binding were increased in Nur77-deficient BMDMs (Fig. 4G). To substantiate this anecdotal observation, we again performed a genome-wide analysis for all AP-1 targets as determined by our HA-Nur77 ChIP-seq. In BMDMs, Nur77 deficiency results in enhanced accessibility (ATAC-seq) of AP-1 target genes upon LPS stimulation compared to the effect of LPS in WT BMDMs. In addition, H3K27ac ChIP-seq signal and Pol II binding is elevated in BMDM-Nur77-KO cells at these specific sites where Nur77 binds and an AP-1 motif has been identified (Fig. 4H).

In conclusion, these findings indicate that Nur77 acts as a direct regulator at AP-1 target genes, locally suppressing Pol II DNA binding and resulting in less accessible (ATAC-seq data) and decreased activity (H3K27ac data) of enhancers and promoters of AP-1 target genes.

### Nur77 modulates LPS-induced expression of AP-1 transcription factors

**Figure 5.**
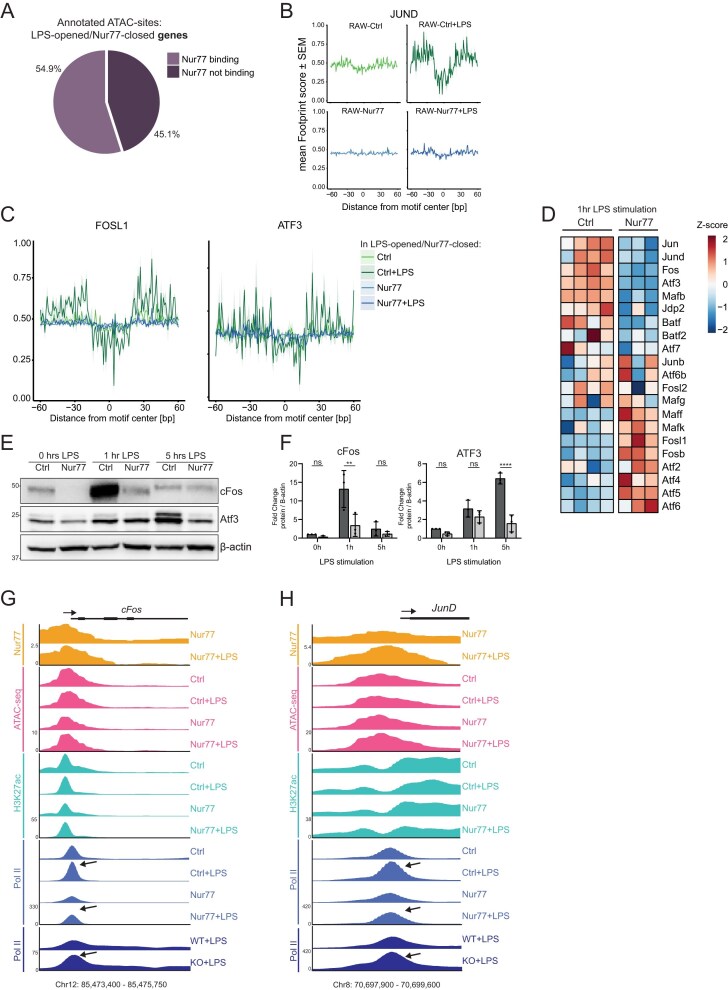
Nur77 represses the transcription of immediate-early genes by reducing Pol II binding but does not affect H3K27ac or chromatin accessibility of these genes. (**A**) Pie chart visualizing the percentage of LPS-opened/Nur77-closed sites as determined by ATAC-seq that are bound by Nur77 as detected in the Nur77 ChIP-seq. (**B**) Aggregate ATAC-seq footprint for *Jund* generated over sites from the LPS-opened/Nur77-closed ATAC-seq group. Data obtained from RAW-Ctrl, RAW-Ctrl+LPS, RAW-Nur77, and RAW-Nur77+LPS are shown in separate panels. The *X*-axis shows the distance in bp to the motif center, *Y*-axis shows the footprinting score ± SEM. (**C**) Aggregate ATAC-seq footprint for *Fosl1* and *Atf3* generated over sites from the LPS-opened/Nur77-closed ATAC-seq group. Data obtained from RAW-Ctrl (light-green), RAW-Ctrl+LPS (dark-green), RAW-Nur77 (light-blue), and RAW-Nur77+LPS (dark-blue) are shown overlaid. The *X*-axis shows the distance in bp to the motif center, *Y*-axis shows the footprinting score ± SEM. **D**: Heatmap depicting the expression of members of the AP-1 family that are expressed in macrophages in RAW-Ctrl and RAW-Nur77 after 1 h of LPS stimulation. Color scale indicates gene expression (z-score). (**E**) Representative western blots showing cFos, and ATF3 protein levels in RAW-Ctrl or RAW-Nur77 cells following 0, 1, or 5 h of LPS stimulation. β-actin was used as a loading control. (**F**) Quantification of independent western blot experiments (*n* = 3) as shown in panel (E) expressed as fold change. ns; non-significant, **P < .01, ****P < .0001 (One-Way ANOVA test). Graphs represent mean ± SD (error bars). Representative example snapshots of Nur77 ChIP-seq (yellow), ATAC-seq (pink), H3K27ac ChIP-seq (turquoise), and Pol II ChIP-seq (purple) at the 5′-end of the *cFos* gene (**G**) or the *JunD* gene (**H**). RNA Pol II snapshots are provided of BMDM-WT and BMDM-Nur77-KO after LPS stimulation (dark-blue). *Y*-axis indicates the sequencing signal in fragments per kB per million reads mapped.

To gain a better understanding of Nur77-mediated gene regulation, we compared our Nur77 ChIP-seq data with the LPS-opened/Nur77-closed sites as defined by ATAC-seq. Unexpectedly, we found that only ~55% of these LPS-opened/Nur77-closed sites are occupied by Nur77 (Fig. 5A). This indicates that the other 45% of LPS-opened/Nur77-closed sites do not show Nur77 recruitment anywhere near those sites, nor at a different place in the gene and, as an example, data for the *Cgref1* gene are shown (Supplementary Fig. S5A). To uncover a second mechanism for Nur77 action in activated macrophages, we looked into transcription factor binding dynamics by employing footprinting analyses on our ATAC-seq dataset [58]. For this a computational tool was applied which enables genome-wide investigation of DNA-protein binding prediction to identify the occupancy of transcription factor motifs [44]. We investigated chromatin dynamics at transcription factor-specific motifs for all ATAC-seq sites. Upon LPS stimulation in RAW-Ctrl cells, for multiple AP-1 family members such as Fos, Jun, and ATF3 an increased occupancy of their motifs was detected (Supplementary Fig. S5B). However, upon overexpression of Nur77, these AP-1 family members have a reduced footprint throughout the genome (Supplementary Fig. S5C). Because of this pronounced detection of AP-1 family members in all our genome-wide analyses, we then investigated the chromatin dynamics specifically at LPS-opened/Nur77-closed sites. Examining JunD motifs, it appears that the footprint score is decreased after LPS stimulation of RAW-Ctrl cells, indicating less accessibility of the motif due to enhanced transcription factor binding under this condition (Fig. 5B, upper right panel). Interestingly, this motif binding seems to be lost in LPS-stimulated cells expressing Nur77 (lower right panel). Similar footprinting patterns were observed for Fosl1 and ATF3 (Fig. 5C), alongside several other AP-1 transcription factors (Supplementary Fig. S5D). This *in silico* prediction of reduced transcription factor binding upon overexpression of Nur77 may be attributed to diminished chromatin accessibility. However, this also raises the possibility that the protein levels of these specific transcription factors are reduced. To explore this scenario of direct regulation of the expression of AP-1 factors, we revisited our RNA-seq dataset at 1 h of LPS stimulation. We observed reduced expression of multiple AP-1 transcription factors upon Nur77 overexpression, including *Jun, Jund, cFos, Atf3/7, Mafb, Jdp2, Batf*, and *Batf2* (Fig. 5D). Of note, other AP-1 factors are not affected by Nur77 overexpression or their expression is increaced (Fig. 5D). Deficiency of Nur77 in BMDMs instead leads to upregulation of *Atf3/5, Jund, Mafg/k, Batf3* and *cFos* expression at 1 h LPS stimulation (Supplementary Fig. S5E). Consistently, protein expression of ATF3 and cFos is diminished in RAW-Nur77 compared to RAW-Ctrl cells both under non-stimulated and LPS-stimulated conditions (Fig. 5E-F). This raises the question how Nur77 regulates the expression of AP-1 genes. Analysis of our ChIP-seq data unveiled prominent Nur77-specific peaks along the gene encoding *Atf3*, accompanied by Nur77-motifs distributed throughout the gene (Supplementary Fig. S5F). In contrast, relatively low Nur77 recruitment was observed at most other AP-1 family members, such as *Jund* and *cFos* (Fig. 5G-H). For most AP-1 genes, no Nur77-motifs were identified, making *Atf3* an exception. These relatively low and dispersed binding profiles for Nur77 may suggest that Nur77 interacts with regulatory complexes or other proteins associated with these immediate-early genes. As indicated by our RIME data, Nur77 interacts with other co-regulators to modulate gene transcription. To determine whether Nur77 indeed also downregulates Pol II binding on these genes, we present Pol II and H3K27ac ChIP-seq and ATAC-seq data for several of these early genes (Fig. 5G-H). At *cFos* and *JunD*, only Pol II binding recruitment is significantly downregulated upon Nur77 overexpression, with almost no change in ATAC-seq or H3K27ac. In contrast, Pol II binding is increased in stimulated Nur77-deficient BMDMs compared to Pol II binding in stimulated WT BMDMs (Fig. 5G and H; in dark-blue). Collectively, these observations suggest that Nur77 suppresses the expression of immediate-early AP-1 transcription factors, most likely mainly through regulation of Pol II activity at these genes.

In conclusion, we propose that Nur77 acts as a regulator of AP-1 target genes by repressing the expression of a number of these upstream AP-1 transcription factors themselves. Our data suggest that Nur77-mediated partial inhibition of AP-1 family expression may explain the downstream 50% chromatin closing of the LPS-opened/Nur77-closed AP-1 sites that lack direct Nur77 binding.

### Nur77 requires its DNA-binding domain to execute an anti-inflammatory function via AP-1 factors

**Figure 6.**
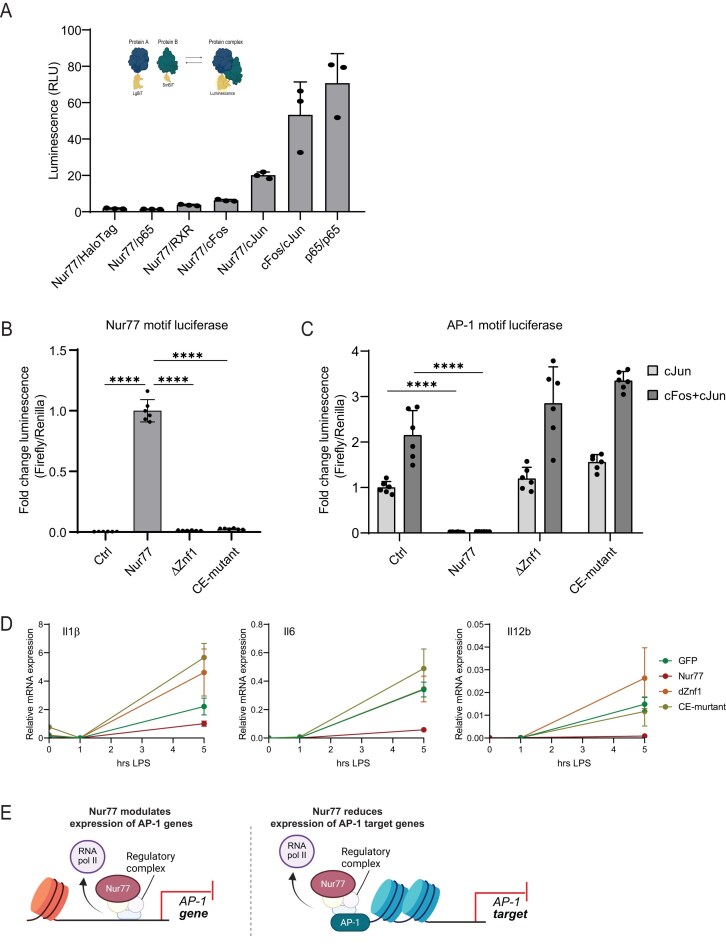
Nur77 repression of AP-1 activity and macrophage inflammation requires an intact Znf1 in its DNA-binding domain. (**A**) Split-luciferase assay to assess protein–protein interaction between Nur77 and RXR, RelA/p65, cFos and cJun. Halotag is included as negative control, and interaction between cFos/cJun heterodimers and p65 homodimers served as positive controls. (**B**) Luciferase assay to determine the transcriptional activity of Nur77(-variants) with a reporter-plasmid containing a Nur77-motif in HEK293T cells. Either Nur77 or Nur77-ΔZnf1, the Nur77-variant lacking the first zinc finger of the DNA-binding domain, or Nur77-CE-mutant were overexpressed and luciferase activity was determined. An empty expression vector was used as a background control and *Renilla* luciferase was co-transfected as a control for transfection efficiency. (**C**) Luciferase assay with a reporter-plasmid containing an AP-1 motif in HEK293T cells. cJun or cFos/cJun were overexpressed together with Nur77 or Nur77-mutants and cJun/cFos activity was measured with an AP-1 luciferase reporter. An empty expression vector was used as a control and *Renilla* luciferase was co-transfected as a control for transfection efficiency. (**D**) The mRNA expression of *Il1β, Il6*, and *Il12b* was determined by qPCR in RAW264.7 cells with inducible expression of Nur77, Nur77-ΔZnf1, or CE-mutant after 1 and 5 h of LPS stimulation. (**E**) A schematic representation of the dual-repression mechanism by which Nur77 represses AP-1 targets. Right panel: Nur77 acts as a regulator of gene expression of AP-1 target genes by binding AP-1, reducing chromatin accessibility, inhibiting H3K27ac, and removing Pol II from the DNA. Left panel: Nur77 modulates expression of several AP-1 genes, repressing their transcription involving reduced Pol II coverage. All graphs represent mean ± SD (error bars); ****P < .0001 (Unpaired *t*-test).

The limited presence of Nur77-motifs (NBRE and NurRE) found enriched at Nur77 sites proximal to AP-1 (target) genes in activated macrophages remains remarkable. This raises the question, even though we did not identify AP-1 factors in our RIME experiment, whether direct protein–protein interaction is necessary for Nur77 to repress expression of AP-1 target genes. We performed split-luciferase experiments that allow detection of protein–protein interaction in live cells. As expected, Nur77-RXR dimerization was identified, however, the interaction of Nur77 with RelA/p65 was not detected (Fig. 6A). In this assay, we observed interaction of Nur77 with cJun and cFos. As positive controls, we incorporated cJun/cFos heterodimerization and p65 homodimerization in these experiments and as a negative control Nur77 together with the Halotag-construct. Taken together, and in line with published data [59, 60], we observed interaction of Nur77 with cJun and cFos. This indicates that the split-luciferase assay, with overexpression of both Nur77 and other factors, is more sensitive than the RIME experiment in RAW267.4 cells, and that the interaction between Nur77 and AP-1 factors seems more stable than with RelA/p65. Next, we aimed to assess the relative importance of Nur77 transcriptional activity in the inhibition of AP-1 target genes. We generated a Nur77-variant lacking the first zinc finger of its DNA-binding domain (Nur77-ΔZnf1) or with mutations in the first zinc finger, Nur77-CE284-285AA (Nur77-CE-mutant), rendering it incapable of DNA-binding but maintaining its nuclear localization signals in the second zinc finger of the DNA-binding domain. The lack of transcriptional activity of Nur77-ΔZnf1 and Nur77-CE-mutant was confirmed in luciferase assays with a reporter-plasmid containing the Nur77-motif showing the absence of a luciferase signal for Znf1-mutants (Fig. 6B). Next, we investigated whether Znf1 of the DNA-binding domain is involved in Nur77-mediated repression of AP-1 activity. We monitored transcriptional activity of c-Jun and c-Fos using an AP-1 luciferase-reporter plasmid. Nur77 inhibits the transcriptional activity of both cJun and cJun/cFos almost completely, whereas Nur77-ΔZnf1 and Nur77-CE-mutant no longer inhibit AP-1 activity (Fig. 6C). Based on these data, we conclude that Nur77 requires Znf1 of its DNA-binding domain to inhibit AP-1 activity. To further dissect Nur77-mediated resolution of inflammation, we generated RAW264.7 cells with inducible expression of Nur77-ΔZnf1 and Nur77-CE-mutant. Overexpression of Nur77 reduces LPS-induced expression of inflammation-related genes such as *Il1β, Il6* and *Il12b*, however, the Nur77-variants with an incomplete or mutated DNA-binding domain no longer inhibit expression of these genes (Fig. 6D).

These data may indicate that Nur77 mediates its anti-inflammatory action in macrophages to some extent through inhibition of the expression of AP-1 target genes. This is in contrast with common statements in literature emphasizing only the connection between Nur77 and NF-kB [48, 51]. Based on our findings, we propose that Nur77 exerts a dual-repression mechanism to limit the expression of inflammatory genes regulated by AP-1 signaling. The first mechanism we described identifies Nur77 as a regulatory factor that binds around AP-1 motifs thereby delimiting expression of these AP-1 target genes (Fig. 6E; right panel). The second mechanism concerns a more indirect Nur77-mediated repression of AP-1 target genes where Nur77 acts as a regulator of expression of AP-1 genes themselves thereby suppressing their downstream targets (Fig. 6E; left panel).

Taken together, our data reveal that in macrophages Nur77 acts on the inflammatory AP-1 signaling cascade by both regulating expression of AP-1 factors themselves and on their targets to maximize resolution of the inflammatory response.

## Discussion

Nur77 deficiency has been demonstrated to result in a pro-inflammatory phenotype in macrophages [19, 20, 26, 46]. Notably, its impact on inflammatory cytokines and mitochondrial metabolism has been identified as pivotal in modulating inflammation [26]. Moreover, Nur77 deficiency exacerbates inflammatory conditions such as atherosclerosis, rheumatoid arthritis, inflammatory bowel disease, and encephalomyelitis, and this phenotype mainly attributes to its function in macrophages [16, 19–22]. The relevance of Nur77 in controlling inflammation positions it as a noteworthy target for treating chronic immunopathology. However, so far, no specific endogenous ligands have been identified for Nur77 [61]. So far, signaling pathways associated with Nur77 in activated macrophages remain poorly understood. We studied Nur77 in macrophages by applying gain- and loss-of-function strategies. Our investigations confirm the anti-inflammatory role of Nur77, but also delineate a dual-mode mechanism through which Nur77 represses cytokine expression (Fig. 6E). First, Nur77 recruitment to chromatin is found enriched at AP-1 motifs in the proximity of AP-1 target genes, as evidenced by ChIP-seq binding signals. Second, Nur77 regulates expression of AP-1 genes, thereby affecting the binding of these transcription factors to their target genes. We observed reduced transcriptional priming, and less accessible chromatin around AP-1 target genes upon overexpression of Nur77. Interestingly, our ChIP-seq data suggest that to modulate the expression of inflammatory genes, Nur77 does not primarily bind directly to the DNA on Nur77 motifs but rather associates with DNA via protein–protein interactions. Moreover, Nur77 associates with Pol II regulatory proteins, which may reduce recruitment of Pol II at immediate-early genes and at AP-1 target genes.

The exact underlying and essential protein–protein interactions of Nur77 to mediate its anti-inflammatory function were elusive and required further investigation. A prior study has also investigated the inhibitory role of Nur77 on the expression of AP-1 family member genes, albeit in cancer cells [57]. It was shown that Nur77 binds to and regulates Pol II at immediate-early genes. In contrast to our findings, these authors showed that Nur77 binding leads to the sequestering of Pol II on the gene body [57], whereas we demonstrate that Nur77 presence in addition leads to less recruitment of Pol II at transcription start and termination sites. Another discrepancy with our study is that we applied Nur77 mutations that only affect Znf1 rather than deleting the entire DNA-binding domain, which is relevant because the second zinc finger of the Nur77 DNA-binding domain contains essential nuclear localization signals. Complete deletion of the DNA-binding domain will result in cytoplasmic localization and interfere with the nuclear function of Nur77 [62].

Taken together, we introduce a novel perspective on Nur77, highlighting that its role in the repression of inflammation in macrophages appears to involve its DNA-binding domain.

Previous studies have predominantly focused on Nur77’s influence on NF-kB activity in inflammatory settings. [48, 51]. Our findings reveal that Nur77 overexpression promotes an anti-inflammatory phenotype, characterized by reduced secretion and mRNA expression of pro-inflammatory cytokines in macrophages. Applying our unbiased approach, we demonstrated that Nur77 strongly suppresses AP-1 factors rather than NF-kB. This observation prompts a reevaluation of the significance of Nur77’s impact on NF-kB. Our study may shift the focus from Nur77’s interaction with NF-kB to its interaction with AP-1 in the context of inflammation, shedding new light on the regulatory mechanisms underlying inflammatory responses in macrophages. Some previous studies have already suggested a role for Nur77 in AP-1 signaling. In human umbilical vein endothelial cells, Nur77 has been shown to inhibit expression of endothelin-1 (ET-1) involving protein–protein interaction of Nur77 with c-Jun. This interaction at the c-Jun promoter was described to decrease c-Jun expression, explaining the Nur77-mediated downregulation of ET-1 expression [63]. Furthermore, extensive studies in tumor-infiltrating T cells revealed that Nur77 plays a crucial role in T cell dysfunction involving AP-1 modulation [64, 65] Also in these cells, Nur77 is present near AP-1 motifs and represses downstream AP-1 effector-gene expression. As an underlying mechanism, it is shown that Nur77 does not affect cJun or cFos expression but inhibits the activity of these transcription factors [64]. In contrast, our data in macrophages reveal that the reduced binding of AP-1 factors to their target genes most likely involves both Nur77-mediated repression of expression of several members of the AP-1 family, as well as inhibition of AP-1 activity at the promoters of target genes. Additionally, there is evidence for a feedback loop where AP-1 factors either activate or inhibit Nur77 expression, indicating a bidirectional regulatory mechanism [59, 66]. More recently, Jiang *et al*. demonstrated that Nur77/NR4A1 inhibits the transcriptional activity of c-Fos in breast cancer cells [60]. In these studies, an interaction between Nur77 and c-Fos and cJun was detected in regular immunoprecipitation experiments. This setup does not involve cross-linking and co-immunoprecipitation is performed in total cell lysates. RIME, however, only reveals interaction when both proteins are localized within close proximity to each other, which is also the case for split-luciferase assays detecting protein–protein interactions in intact cells. Given that Nur77 is localized in the nucleus, this assay also focuses solely on nuclear Nur77-protein interactions. Understanding AP-1 and Nur77 dynamics in detail is crucial as they highlight the potential of Nur77 to influence inflammatory responses. Future experiments should explore the interaction between AP-1 and Nur77 in macrophages under prolonged chronic stimulation *in vitro* and *in vivo* to further validate these results. An intriguing question raised by our study is how Nur77 connects to the Pol II complex as indicated by our RIME data. An attractive hypothesis is that Nur77 associates with the mediator (MED)-complex, which is supported by the observation that the activity of Nur77 is affected by TRAP220/MED1 [67]. This would be in line with recent structural analyses of the MED-complex revealing that recruitment of certain nuclear receptors modulates the conformation of this complex thereby affecting Pol II binding and activation [68]. However, addressing this question requires extensive studies that go beyond the scope of the current work.

In conclusion, our findings provide a further mechanistic understanding of Nur77’s effect in pro-inflammatory macrophages. Especially the regulation of immediate-early AP-1 transcription factor expression as well as the activity of these factors turn out to play a pivotal role in Nur77-mediated resolution of inflammation.

## Supplementary Material

gkag808_Supplemental_File

## Data Availability

All sequencing raw and processed data generated in this study have been deposited in the Gene Expression Omnibus (GEO) database under the accession number: GSE274016 for RNA-seq in RAW264.7 cells; GSE273992 for ChIP-seq in RAW264.7 cells; GSE273991 for ATAC-seq in RAW264.7 cells; GSE325918 for RNA-seq in BMDM-WT and BMDM-Nur77-KO; GSE326173 for ChIP-seq in BMDM-WT and BMDM-Nur77-KO; and GSE326041 for ATAC-seq in BMDM-WT and BMDM-Nur77-KO. The mass spectrometry proteomics (RIME) data have been deposited to the ProteomeXchnage Consortium via the PRIDE partner repository with the data set identifier PXD054271.
