# Supplementary material for: Nuclear receptor Nur77 resolves the inflammatory response of macrophages primarily by regulating expression of AP-1 transcription factors and their target genes
Source: Nucleic Acids Res. 2026 Aug 17;54(15):gkag808. doi: 10.1093/nar/gkag808 (PMC13478752; doi:10.1093/nar/gkag808)
Supplement: Supplementary file 1 [file gkag808_supplemental_file.pdf]

Supplemental Figure S1

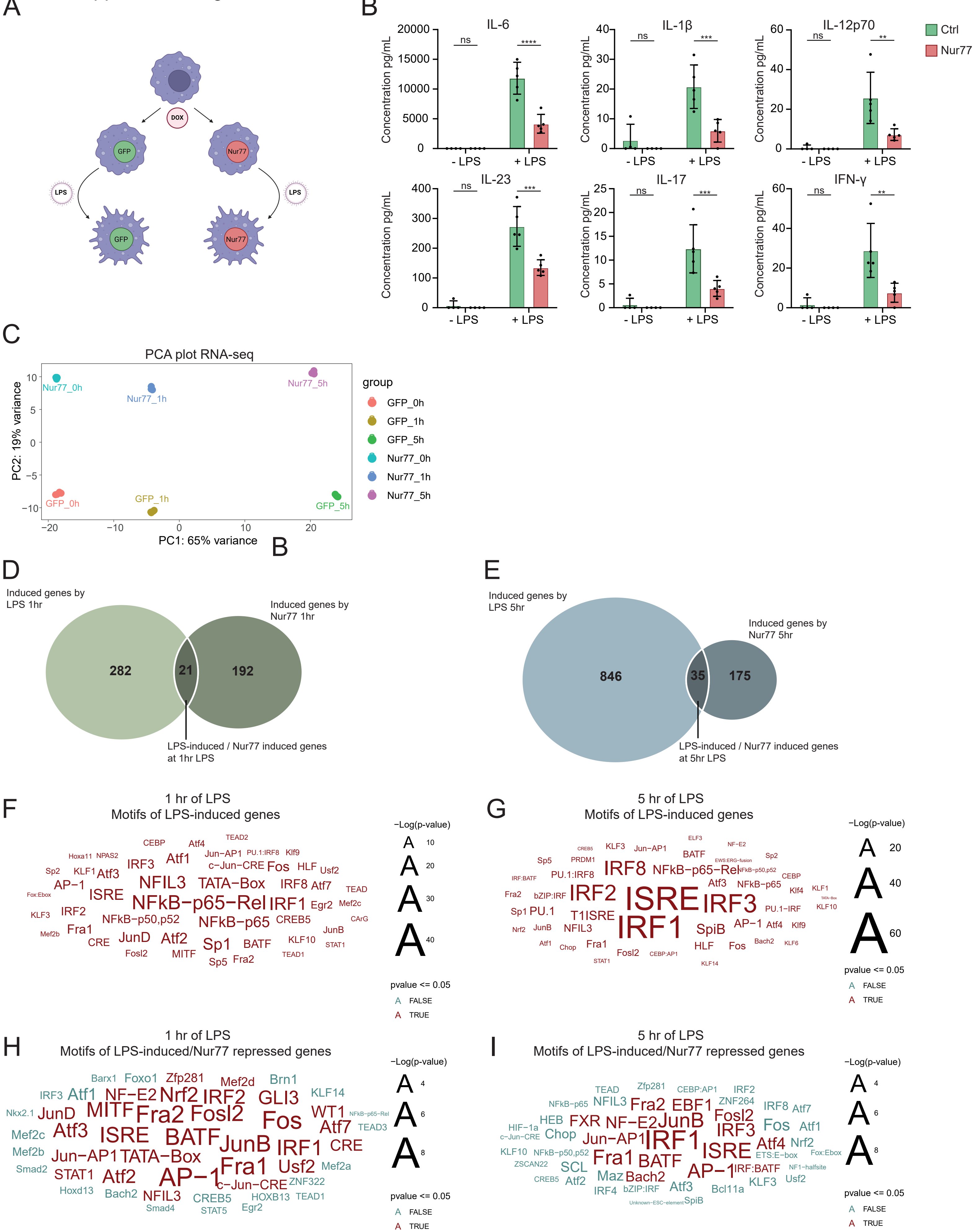

Supplemental Figure S1, continued

J

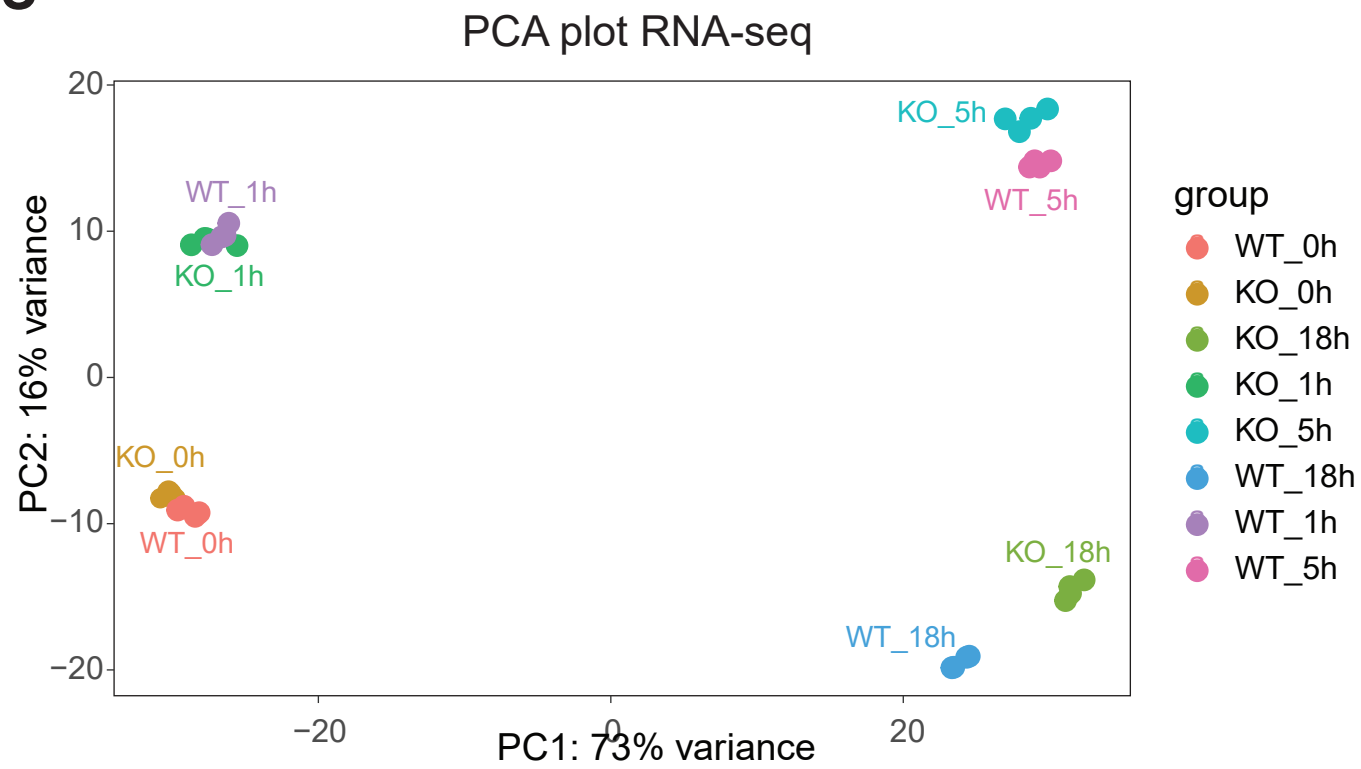

K

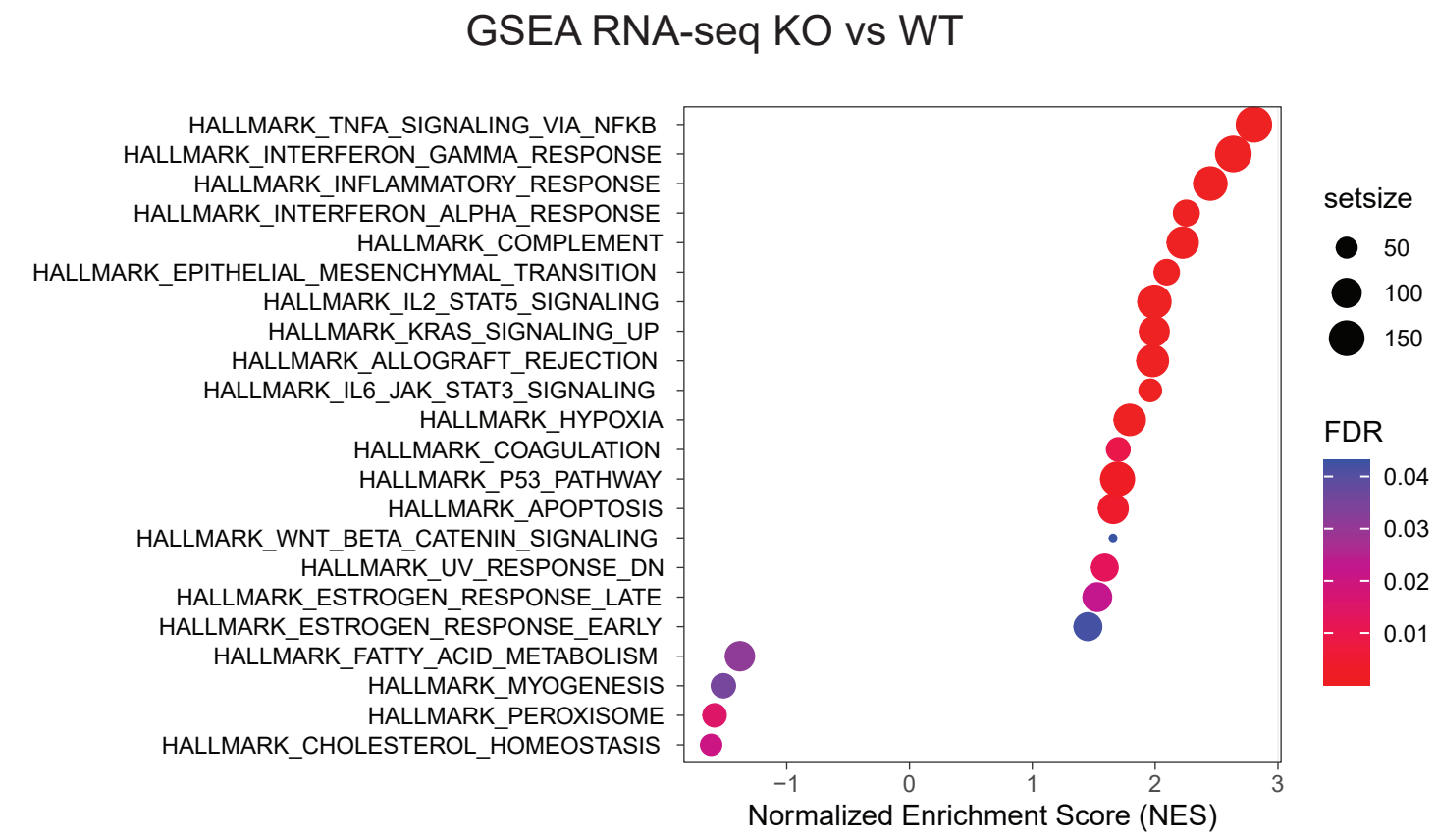

L

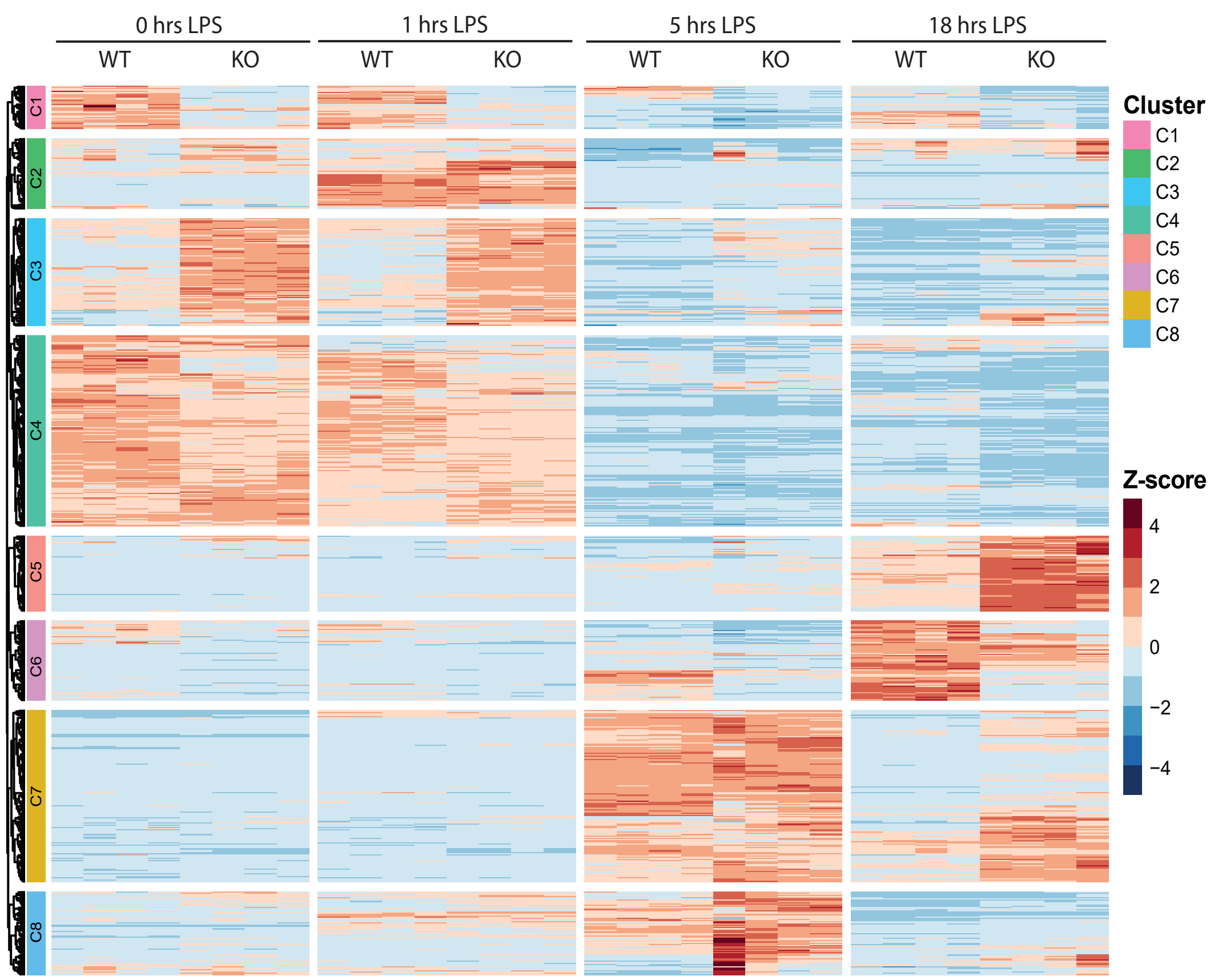

M

Supplemental Figure S1, continued

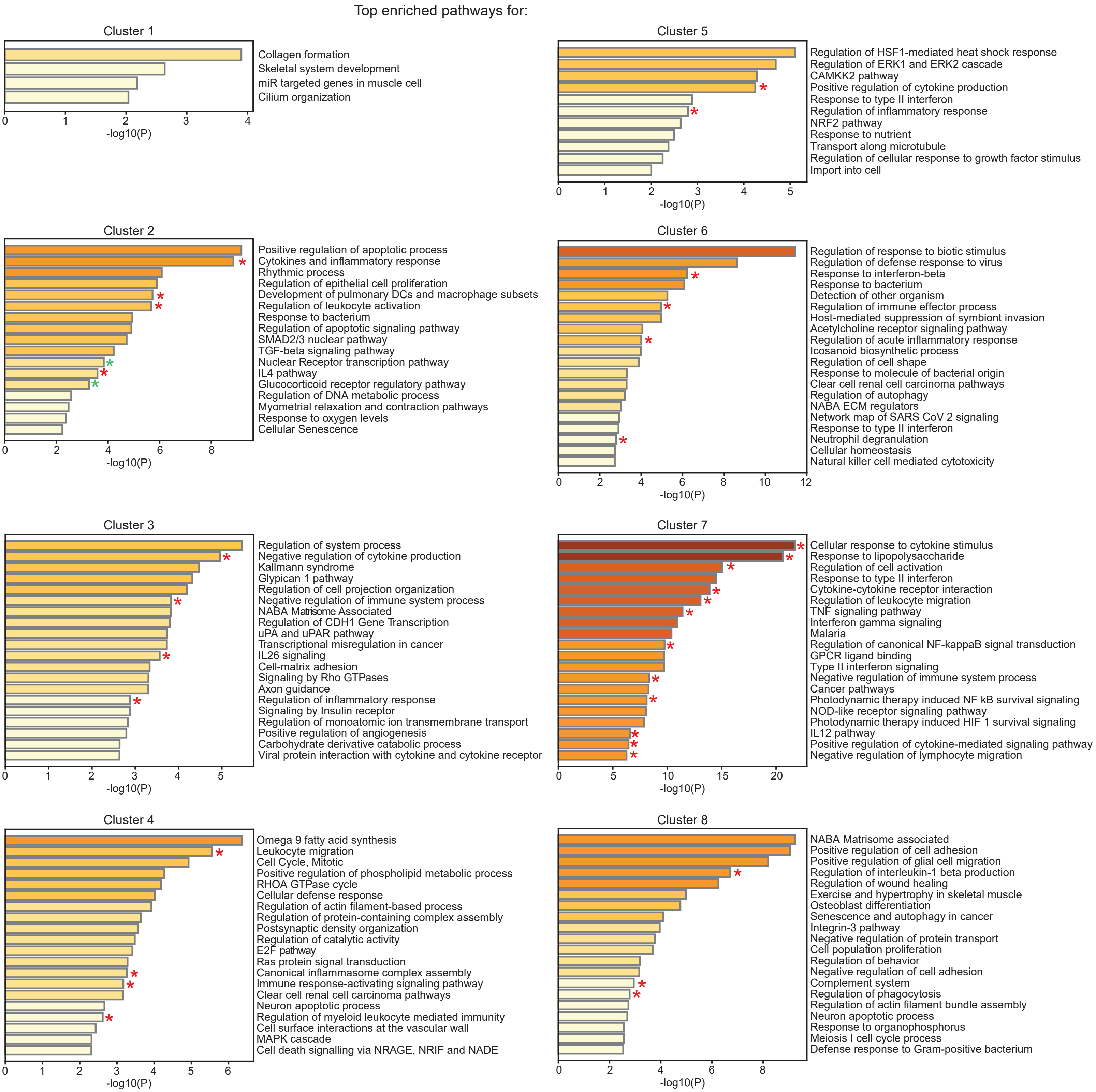

N

Supplemental Figure S1, continued

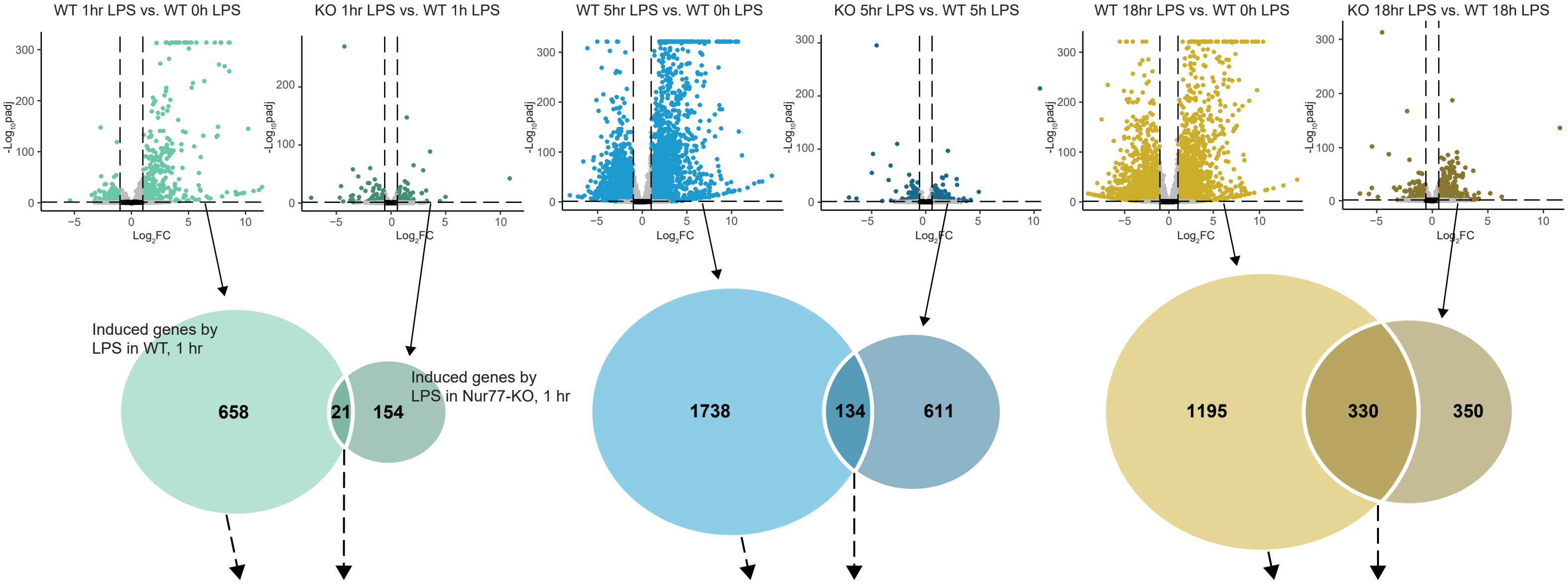

Motif	Motif
NFκB-p65	JunB
NFκB-Rel	Atf3
TATA-Box	BATF
NFκB2-p52	Fra2
NFκB-p50	AP-1

NFκB-related TFs

AP-1 TFs

Motif	Motif
ISRE	Znf46
IRF1	Bapx1
IRF2	Atf3
IRF3	JunB
IRF8	Znf415

Motif	Motif
ISRE	ISRE
IRF3	IRF1
IRF2	IRF2
IRF1	IRF3
IRF8	IRF8

O

Supplemental Figure S1, continued

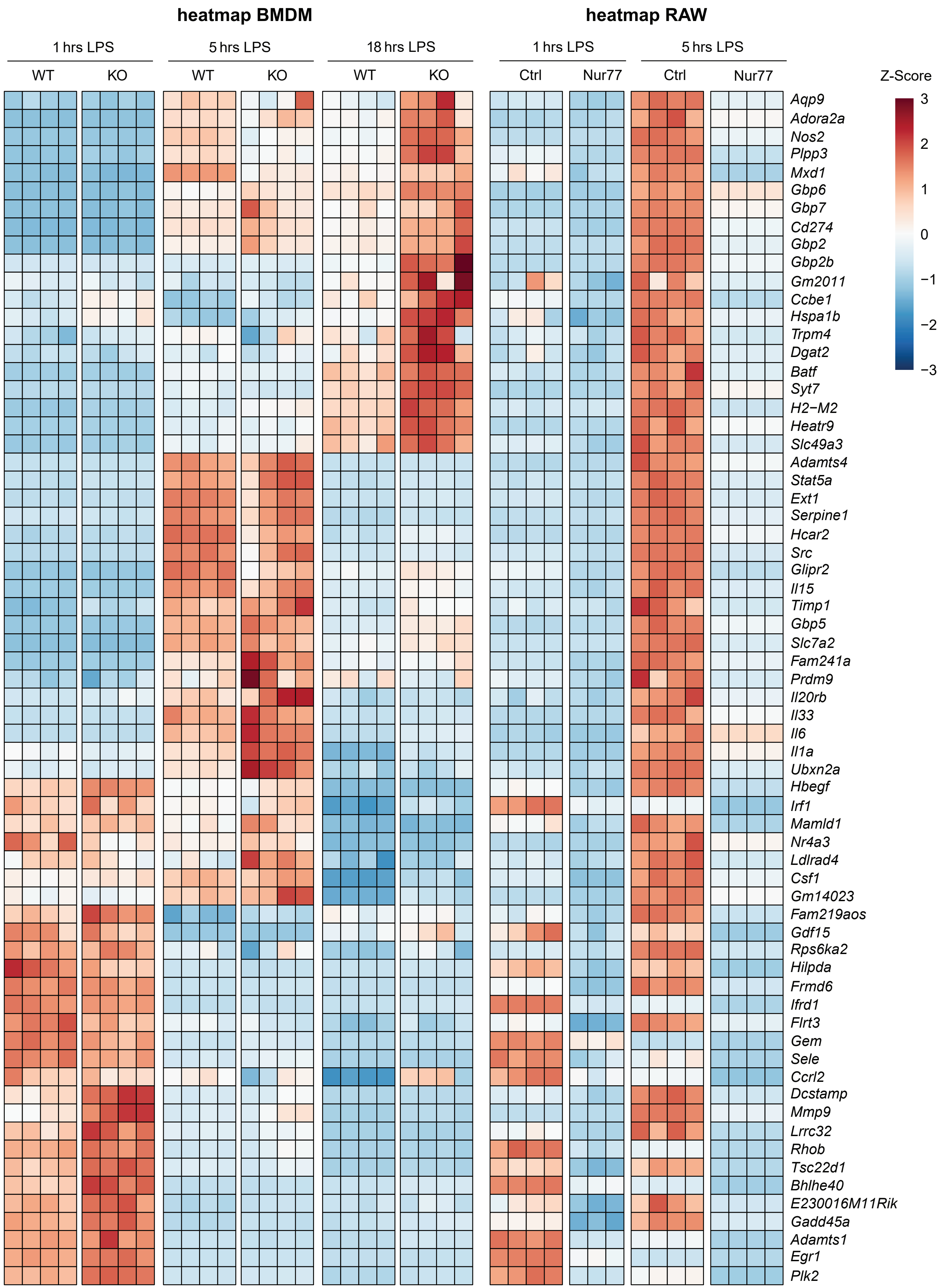

P

Supplemental Figure S1, continued

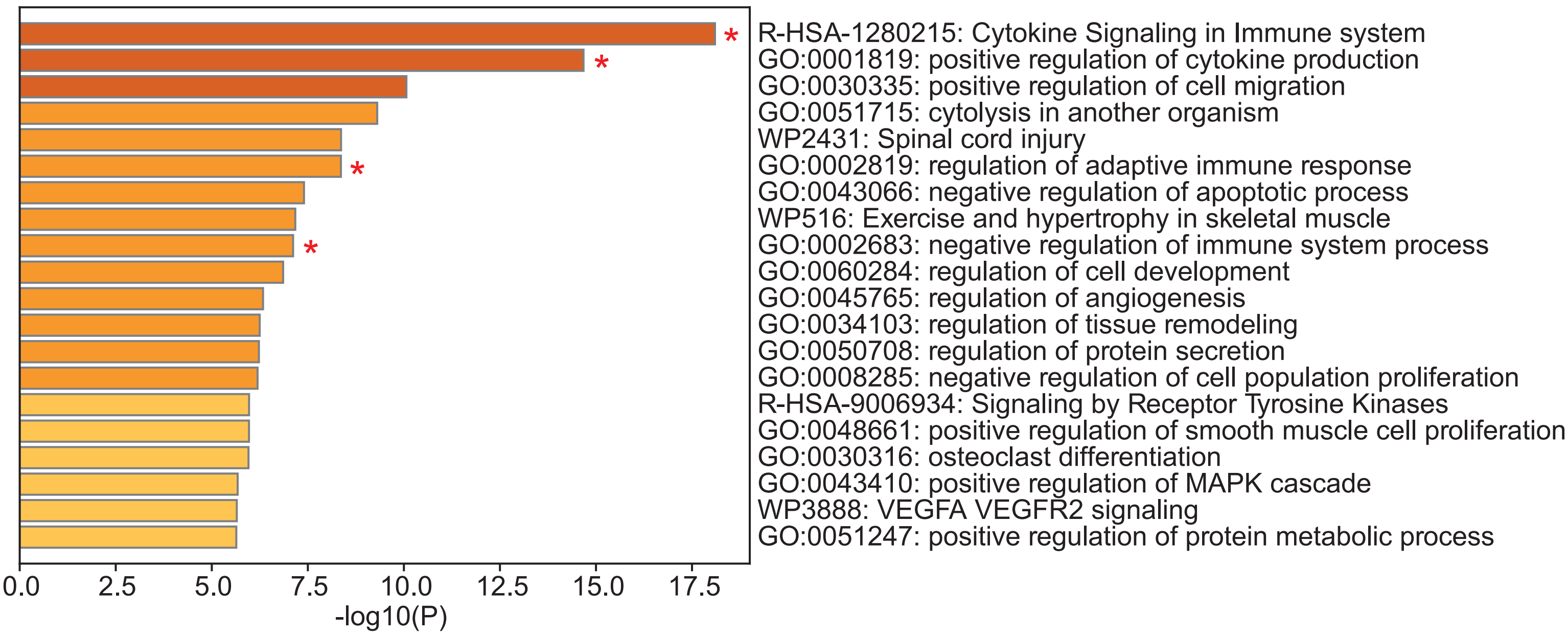

Figure. S1. Analysis of RNA-seq data of RAW-Ctrl and RAW-Nur77, and BMDM-WT and BMDM-Nur77-KO cells

A: Experimental setup in RAW264.7 cells with inducible overexpression of HA-Nur77 (RAW-Nur77) or GFP RAW-Ctrl). Cells were incubated with 50ng/mL doxycycline (DOX) for 18 hours and stimulated with lipopolysaccharies (LPS) to study Nur77 in macrophage inflammation.

B: Protein secretion of IL-6, IL-1β, IL-12p70, IL-23, IL-17, and IFN-γ in the supernatant of RAW-Ctrl and RAW-Nur77 cells before and after 18 hours of LPS stimulation. Data are shown as means ± SDs, n=5. P values were calculated using one-way ANOVA with Tukey's post hoc test. ns non-significant, **P<0.01,***P<0.001,****P<0.0001.

C: Principal component analysis (PCA) plot on conditions of the RNA-seq performed on RAW-Ctrl and RAW-Nur77 cells after 0, 1, or 5 hours of LPS stimulation. Each dot represents one sample condition.

D: Additional analysis of the data shown in Fig. 1C. The overlap between significantly LPS-induced genes in RAW-Ctrl cells (n=303, padj<0.05;FC>1) and Nur77-induced genes after 1 hour of LPS stimulation (RAW-Nur77 compared to RAW-Ctrl after 1 hour of LPS; n=213, padj<0.05;FC>1) is shown by a Venn Diagram. The group of overlapping genes that are LPS-induced/Nur77-induced is shown in dark green (n=21).

E: Additional analysis of the data shown in Fig. 1D. The overlap between significantly LPS-induced genes in RAW-Ctrl cells (n=881, padj<0.05;FC>1) and Nur77-induced genes after 5 hours of LPS stimulation (RAW-Nur77 compared to RAW-Ctrl after 5 hours of LPS; (n=210, padj<0.05;FC>1) is shown by a Venn Diagram. The group of shared genes indicated as LPS-induced/Nur77-repressed is shown in dark blue (n=35).

F: Word cloud showing known-motif enrichment analysis in the promoters of 1 hour LPS-induced genes. The font size represents the –log(p-value) and colors correspond to significance (dark red=p-value ≤ 0.05, blue=p-value > 0.05) factor motifs within 200bp of Nur77 peaks after 1 hour of LPS stimulation versus Nur77 ChIP-seq sites without LPS stimulation.

G: Word cloud showing known-motif enrichment analysis in the promoters of 5 hours LPS-induced genes. The font size represents the –log(p-value) and colors correspond to significance (dark red=p-value ≤ 0.05, blue=p-value > 0.05) factor motifs within 200bp of Nur77 peaks after 1 hour of LPS stimulation versus Nur77 ChIP-seq sites without LPS stimulation.

H: Word cloud showing known-motif enrichment analysis in the promoters of LPS-induced/Nur77-repressed genes after 1 hour LPS. The font size represents the –log(p-value) and colors correspond to significance (dark red=p-value ≤ 0.05, blue=p-value > 0.05) factor motifs within 200bp of Nur77 peaks after 1 hour of LPS stimulation versus Nur77 ChIP-seq sites without LPS stimulation.

I: Word cloud showing known-motif enrichment analysis in the promoters of LPS-induced/Nur77-repressed genes after 5 hours LPS stimulation (right cloud). The font size represents the –log(p-value) and colors correspond to significance (dark red=p-value ≤ 0.05, blue=p-value > 0.05) factor motifs within 200bp of Nur77 peaks after 1 hour of LPS stimulation versus Nur77 ChIP-seq sites without LPS stimulation.

J: Principal component analysis (PCA) plot on conditions of the RNA-seq performed on BMDM-WT and BMDM-Nur77-KO cells after 0, 1, 5 or 18 hours of LPS stimulation. Each dot represents one sample condition.

K: Gene set enrichment analysis (GSEA) of gene expression in BMDM-Nur77-KO over BMDM-WT cells at 18 hours LPS stimulation.

L: Hierarchical clustering of genes and z-score heatmaps of differentially expressed genes between BMDM-WT and BMDM-Nur77-KO at 0, 1, 5, and 18 hours of LPS stimulation as determined by RNA-seq. Color annotation represents the unbiased clustering of 8 groups [Cluster(C)1-8]. Color scale indicates gene expression (z-score).

M: GSEA of genes expressed in BMDM-Nur77-KO over BMDM-WT and grouped in distinct clusters as determined in Figure S1J. Pathways related to inflammatory responses are marked with a red asterisk, and pathways related to nuclear receptor signaling with a green asterisk (*).

N: Scatterplots of RNA-seq data showing 1, 5 and 18 hours LPS-regulated gene expression in BMDM-WT cells and BMDM-Nur77-KO cells. At every time point the overlap between genes significantly induced by LPS in BMDM-WT cells and genes even further induced upon Nur77-deficiency in BMDM cells is shown by Venn Diagrams. The group of overlapping genes that are LPS-induced/Nur77-deficiency induced is shown and a Known-motif analysis is shown in Tables. Significance cutoffs are shown as dotted lines.

O: Heatmaps of 66 genes that were identified in RAW cells as LPS-induced/Nur77-repressed with enhanced expression in LPS-treated Nur77-KO BMDMs compared to LPS-treated WT BMDMs.

P: GSEA of the 66 genes highlighted in Table S1 and characterized in RAW264.7 cells as LPS-induced/Nur77-repressed that also show enhanced expression in LPS-treated Nur77-KO BMDMs compared to LPS-treated WT BMDMs.

Supplemental Figure S2

A

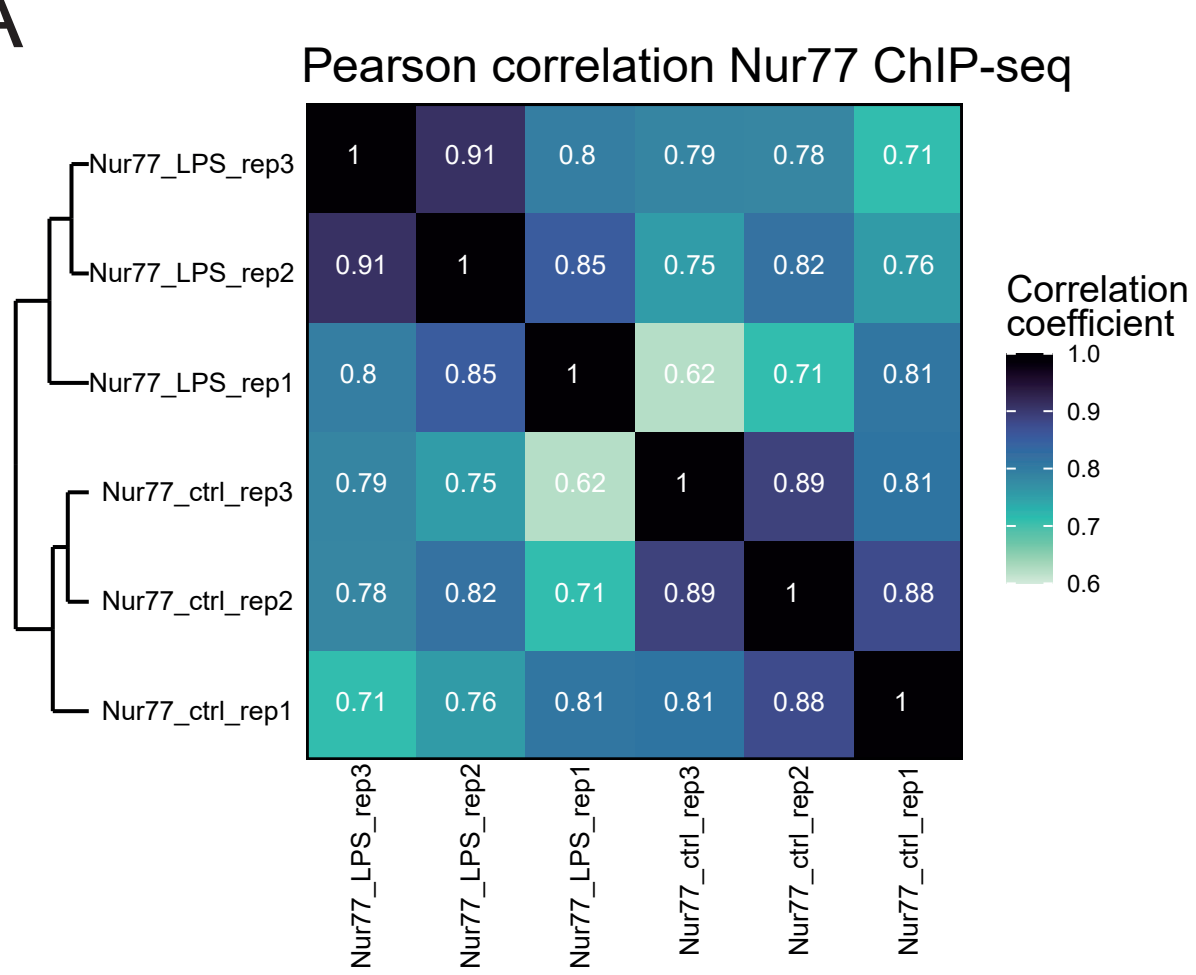

B

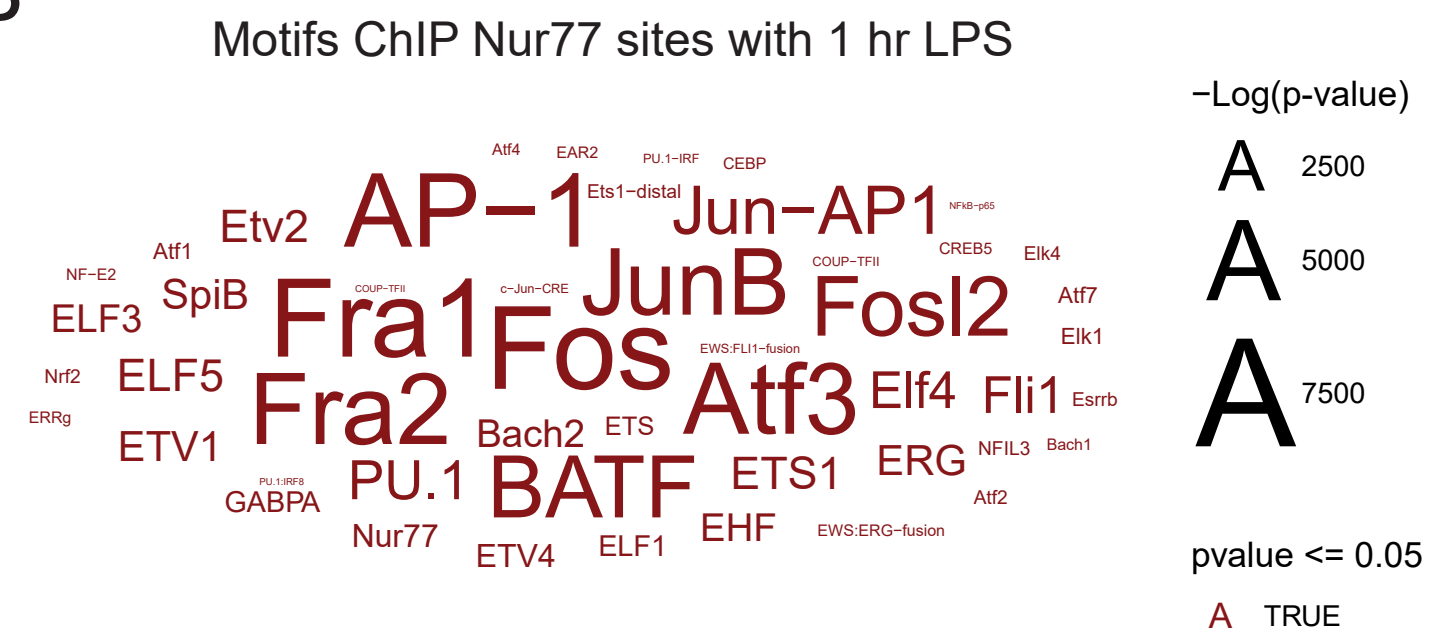

C

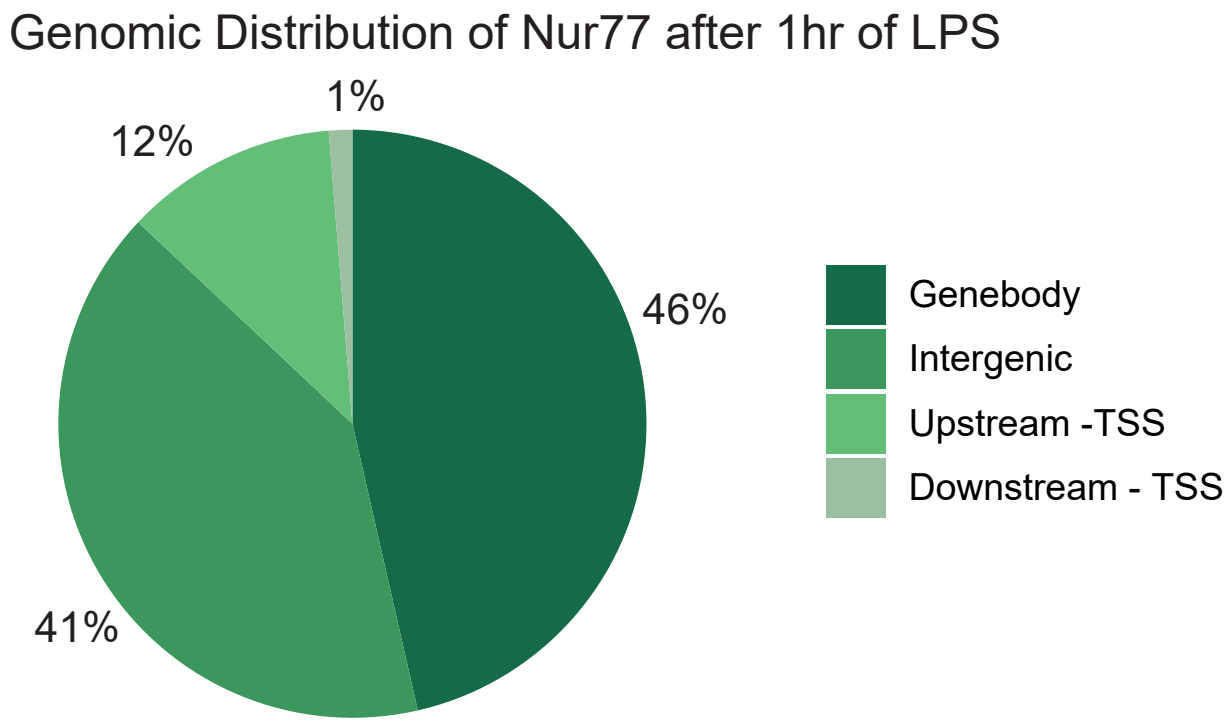

D

Number of Nur77 ChIP-seq peaks

Motif	Ctrl	LPS
Total	29042	24070
NBRE total	4717	3360
NurRE total	264	206
DR5 total	143	110
NBRE unique	4559	3248
NurRE unique	140	113
DR5 unique	107	86
NBRE +NurRE	123	90
NBRE + DR5	35	21
NurRE + DR5	1	2
NBRE + NurRE + DR5	0	1

Figure S2. ChIP-seq of Nur77 in RAW264.7 cells before and after LPS stimulation
A: Correlation heatmap based on Nur77 HA-ChIP-seq data in RAW-Nur77 cells before and after LPS stimulation. Clustering of the samples is based on all called peaks and represents Pearson correlations between individual ChIP-seq samples.
B: Additional data to Figure 2B. In a word cloud known-motif enrichment within 200bp of Nur77 ChIP-seq sites after 1 hour of LPS stimulation is shown. The font size represents the –log(p-value) and colors correspond to significance (dark red=p-value ≤ 0.05).
C: The genomic distribution of Nur77 ChIP-seq peaks after 1 hour of LPS stimulation is shown in a pie chart.
D: Nur77 HA-ChIP-seq peaks in RAW-Nur77 cells were analyzed for NBRE, NuRE and DR5 motifs; see Materials and Methods for details on motifs applied in this search. The number of peaks with these motifs in unstimulated cells (Ctrl) and after 1 hour LPS stimulation (LPS) are presented.

Supplemental Figure S3

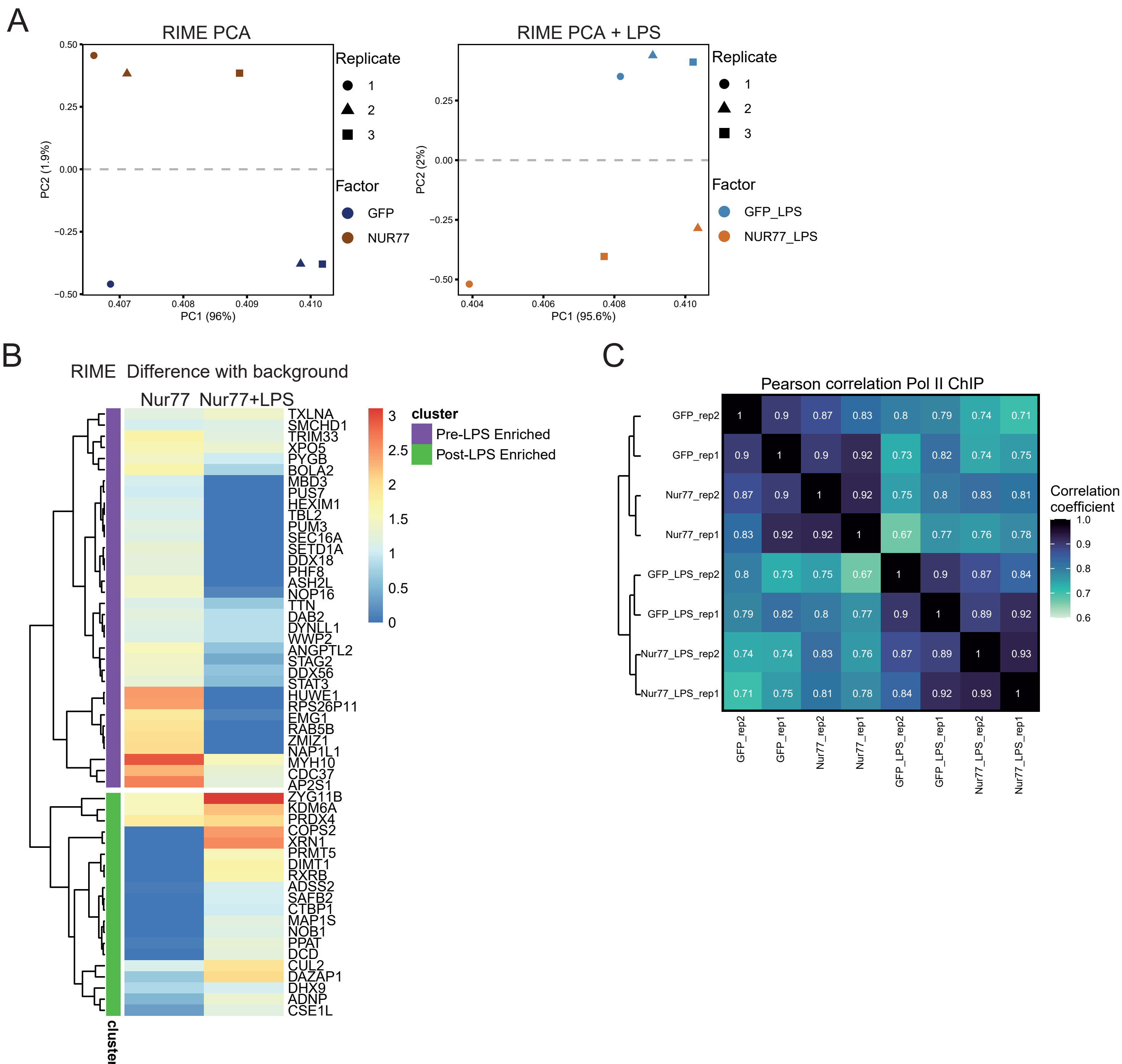

Figure S3. Identification of Nur77 interacting proteins by RIME and Pol II ChIP-seq
A: Principal component analysis (PCA) plot on conditions of the RIME performed on RAW-Ctrl and RAW-Nur77 before (left plot) and after 1 hour of LPS stimulation (right plot). Each symbol represents a different replicate.
B: Heatmap depicting the enrichment of the top hits from the RIME analysis in RAW-Nur77 cells before and after 1 hour LPS stimulation. The color scale indicates the LFQ difference between either RAW-Nur77 against the background or RAW-Nur77+LPS against the background. The proteins that are specifically enriched to bind Nur77 before LPS stimulation are shown in the purple cluster and after LPS stimulation in the green cluster.
C: Correlation heatmap based on Pol II ChIP-seq data from RAW-Ctrl and RAW-Nur77 cells before and after LPS stimulation. Clustering of the samples is based on all called peaks and represents Pearson correlations between individual ChIP-seq samples.

Supplemental Figure S4

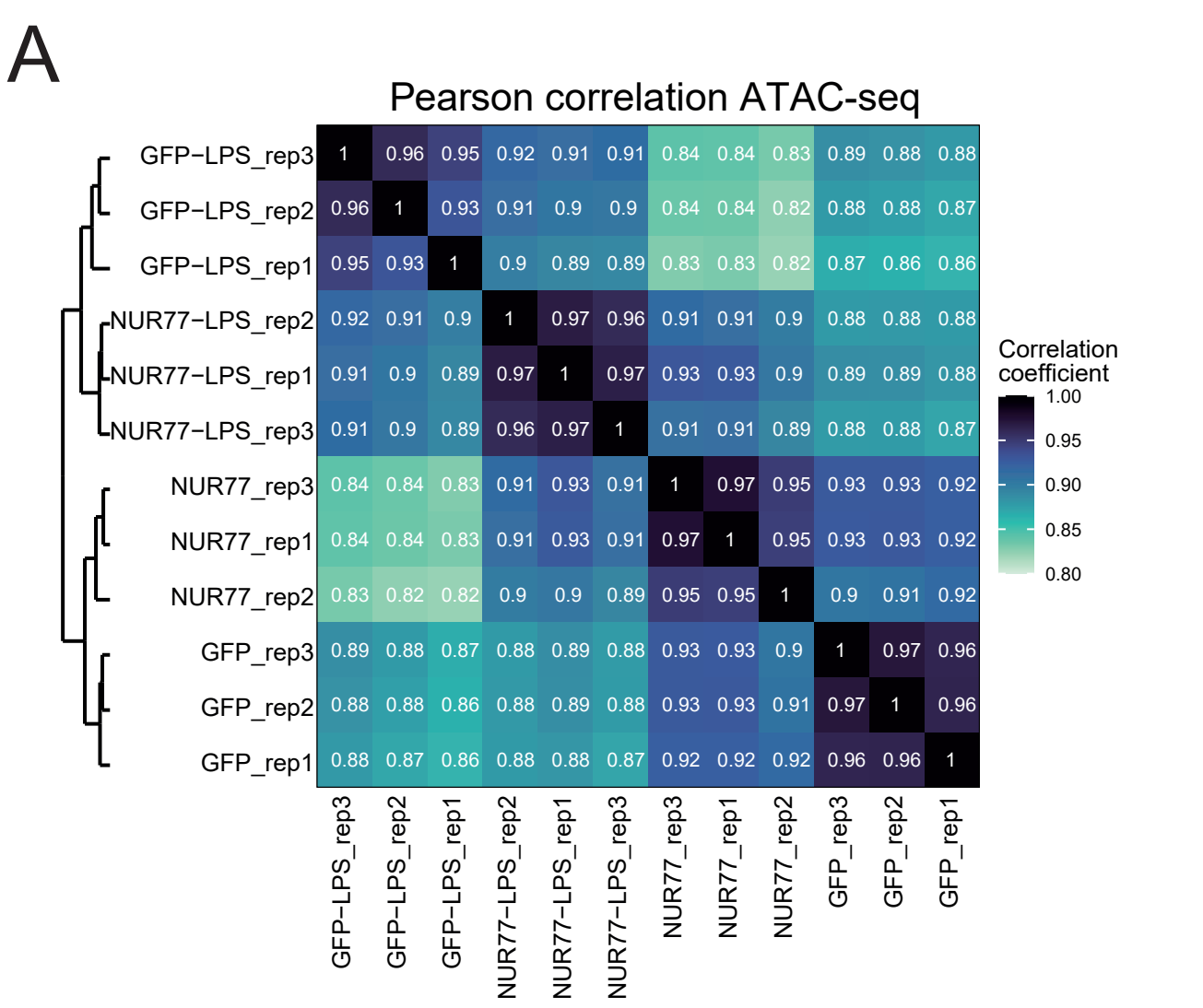

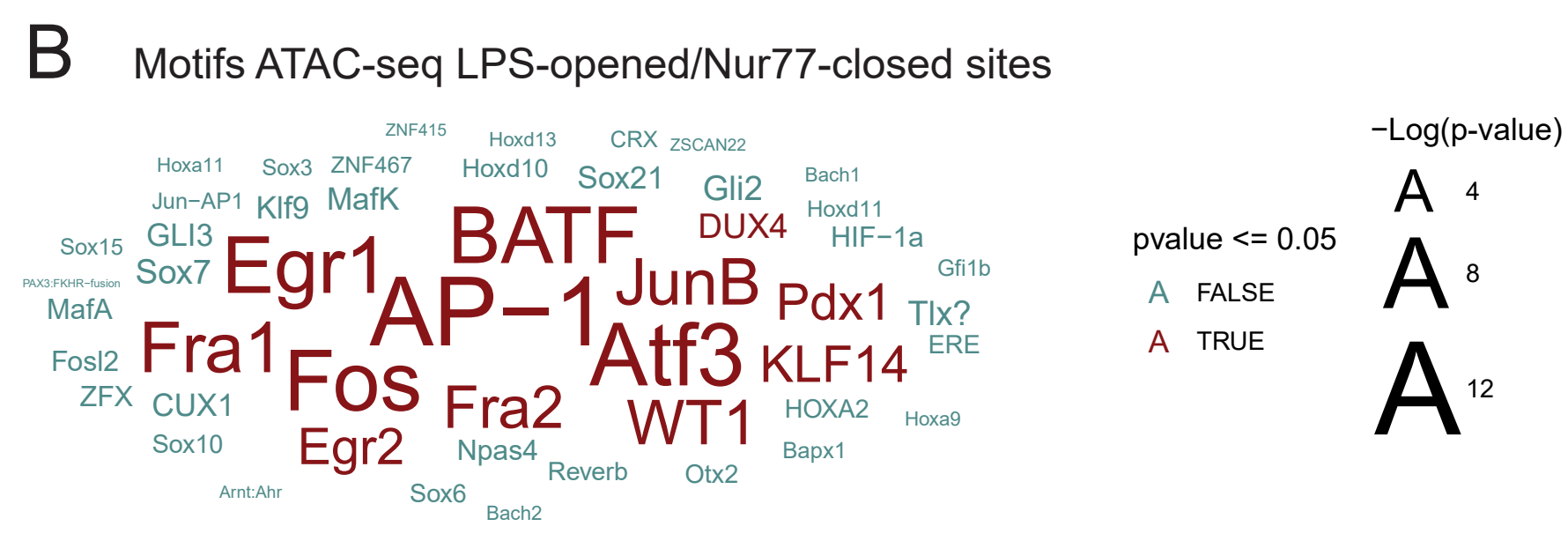

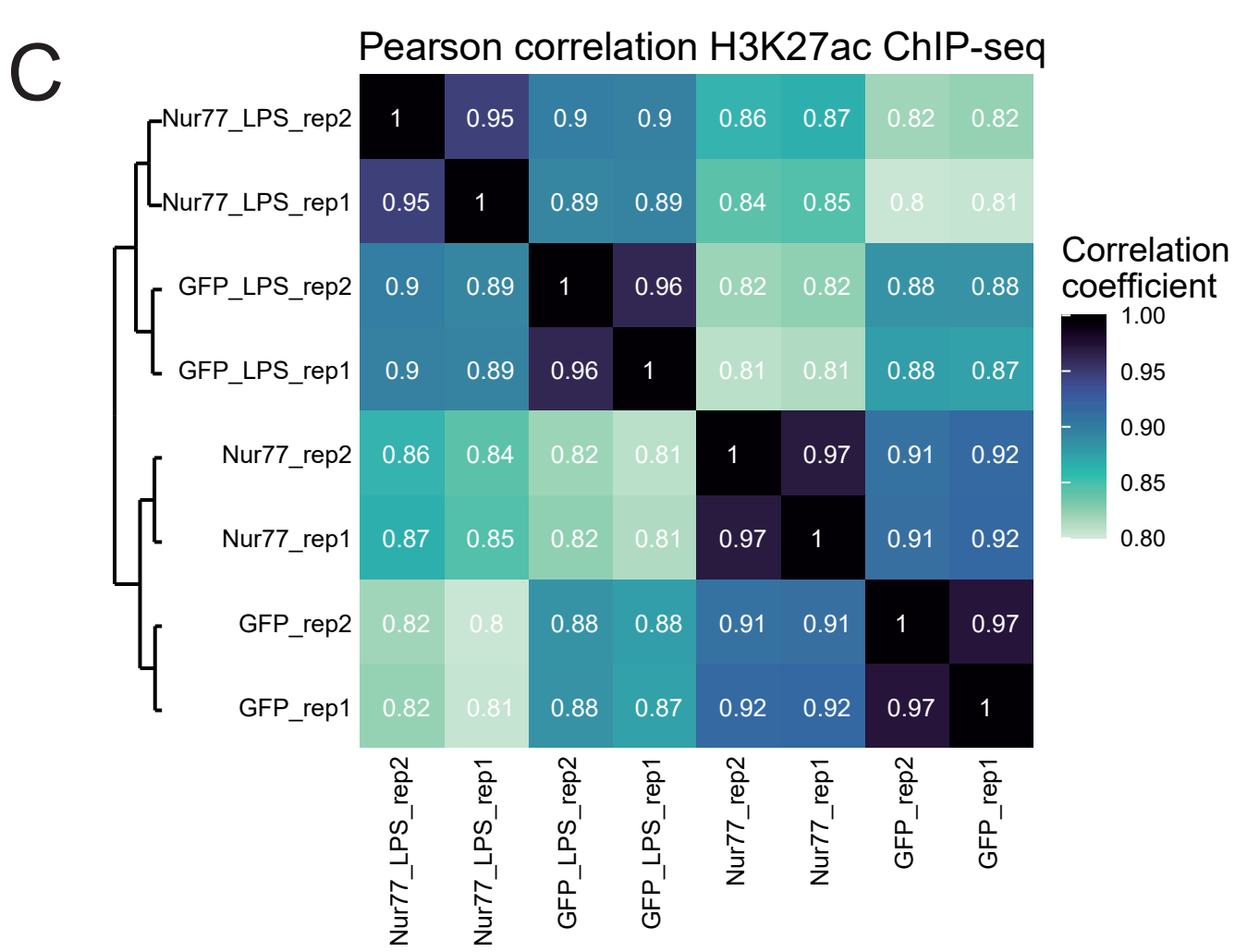

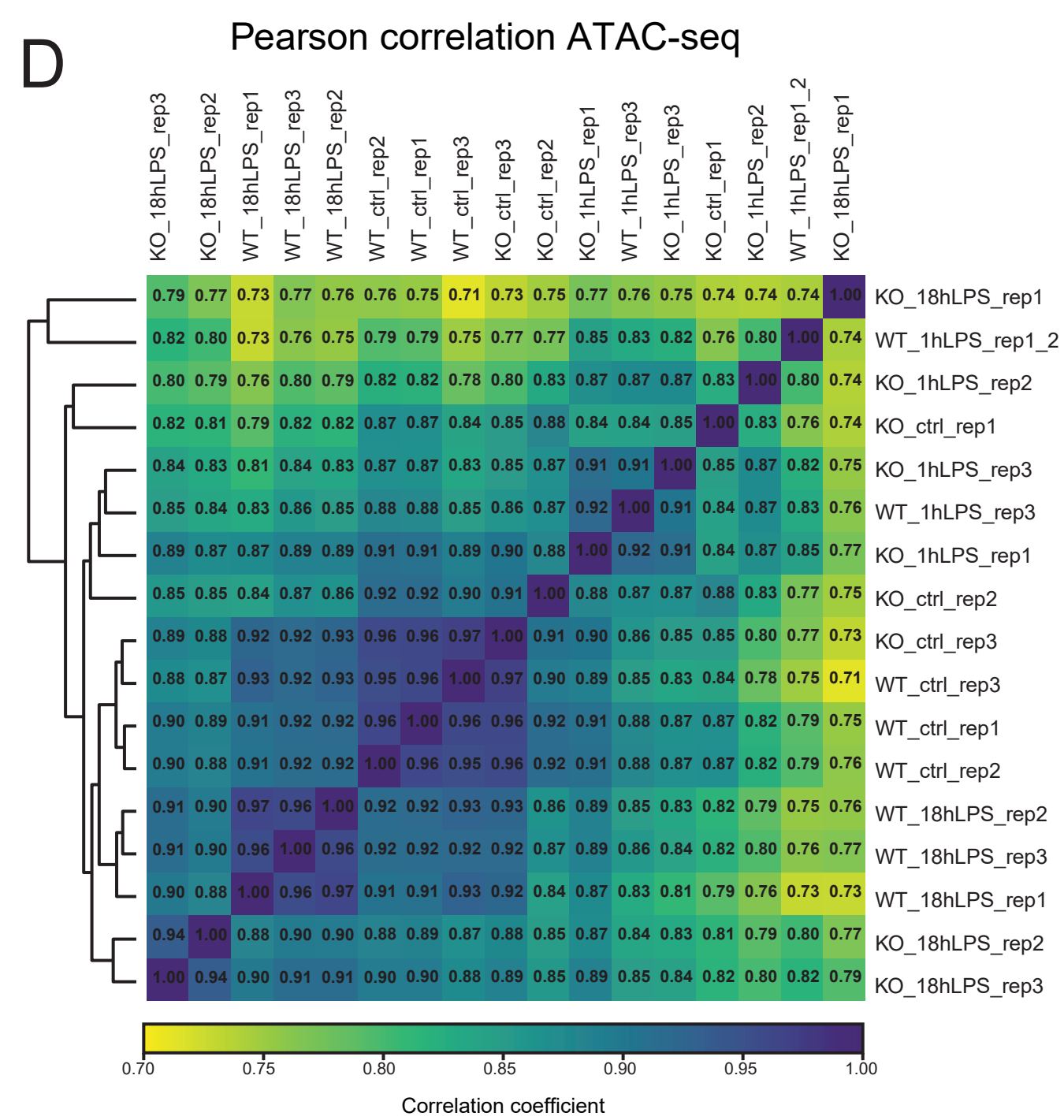

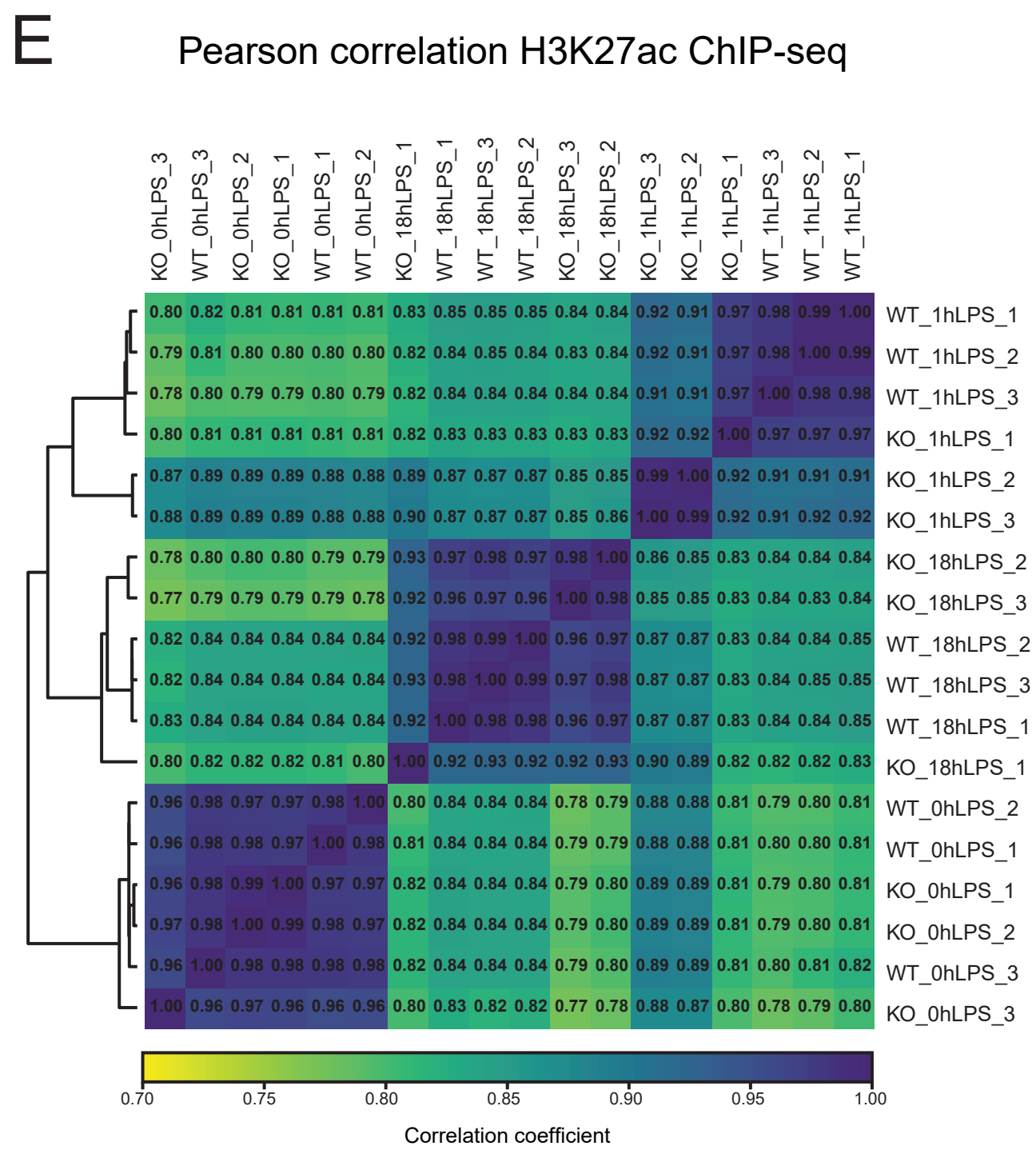

Figure S4. ATAC-seq and ChIP-seq H3K27ac data analysis

A: Correlation heatmap based on ATAC-seq in RAW-Ctrl and RAW-Nur77 cells before and after LPS stimulation. Clustering of the samples is based on all called peaks and represents Pearson correlations between individual ATAC-seq samples.

B: Word cloud showing known motif enrichment analysis within 200bp of ATAC-seq sites of LPS-opened/Nur77-closed genes. The font size represents the –log(p-value) and colors correspond to significance (dark red= p-value ≤0.05, blue= p-value > 0.05).

C: Correlation heatmap based on H3K27ac ChIP-seq data in RAW-Ctrl and RAW-Nur77 cells before and after LPS stimulation. Clustering of the samples is based on all called peaks and represents Pearson correlations between individual ChIP-seq samples.

D: Correlation heatmap based on ATAC-seq in BMDM-WT and BMDM-Nur77-KO cells after 0, 1 and 18 hours of LPS stimulation. Clustering of the samples is based on all called peaks and represents Pearson correlations between individual ATAC-seq samples.

E: Correlation heatmap based on H3K27ac ChIP-seq in BMDM-WT and BMDM-Nur77-KO cells after 0, 1 and 18 hours of LPS stimulation. Clustering of the samples is based on all called peaks and represents Pearson correlations between individual ChIP-seq samples.

Supplemental Figure S5

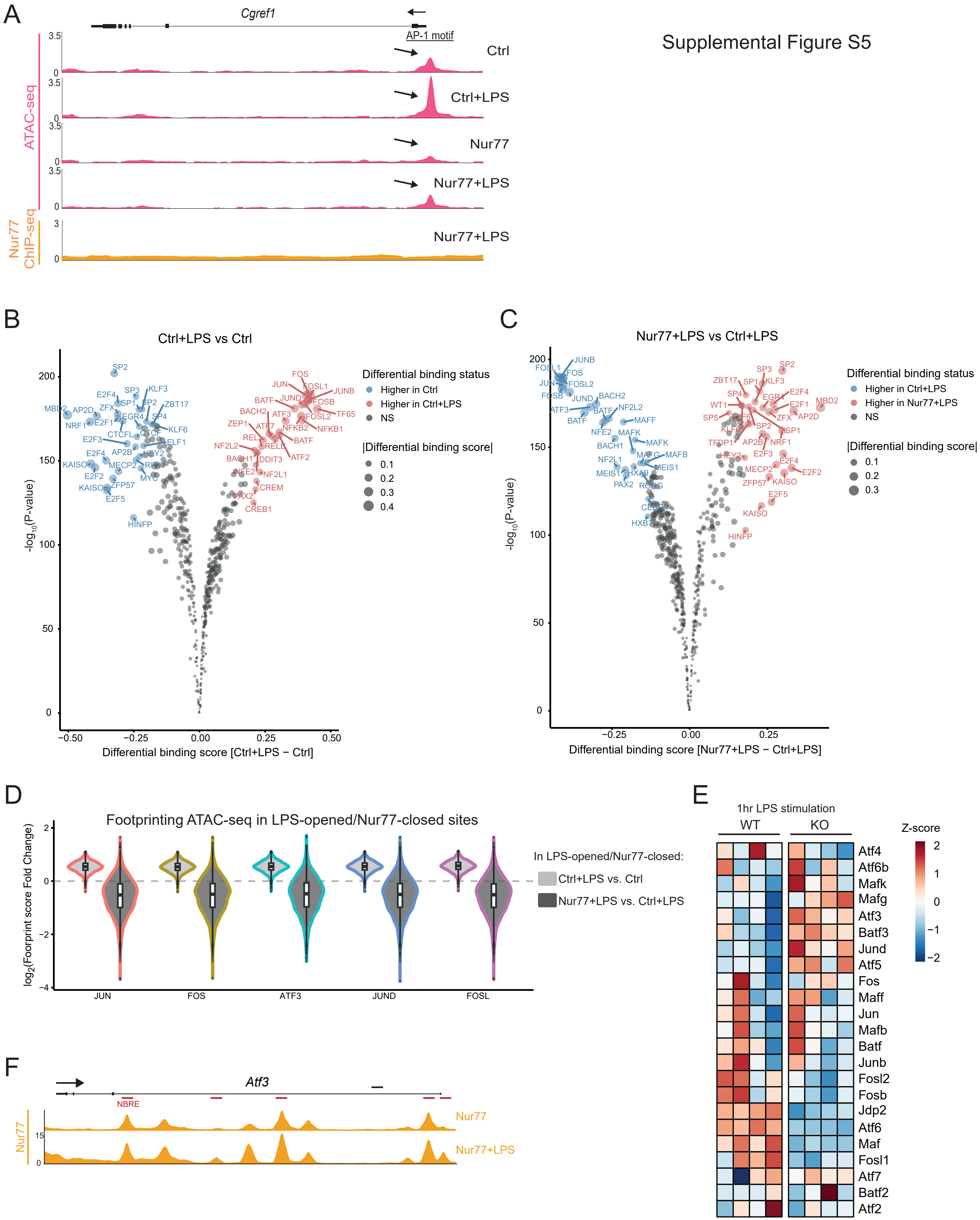

Figure S5. Analyses of ATAC-seq data

A: Representative example snapshot of ATAC-seq (pink) and Nur77 HA-ChIP-seq (yellow) at the Cgref1 gene. The Y-axis indicates the sequencing signal in fragments per kb per million reads mapped. The AP-1 motif is indicated.

B: Scatterplot showing transcription factor (TF) binding to its motif based on footprinting analysis of ATAC-seq data in RAW-Ctrl cells after LSP stimulation versus before LPS stimulation. The X-axis shows the differential binding score of the TFs, the Y-axis shows the p-value of the binding of the TFs. Each dot represents a TF with its known motif (red=higher binding, blue=lower binding in RAW-Ctrl after LPS stimulation versus before). The size of the dot represents differential binding score.

C: Scatterplot showing transcription factor (TF) binding to its motif based on footprinting analysis of ATAC-seq data in RAW-Nur77 cells versus RAW-Ctrl, both after LPS stimulation. The X-axis shows the differential binding score of the TFs, the Y-axis shows the p-value of the binding of the TFs. Each dot represents a TF with its known motif (red=higher binding, blue=lower binding in RAW-Nur77 after LPS stimulation versus RAW-Ctrl after LPS stimulation). The size of the dot represents the differential binding score.

D: Violin plots showing footprinting scores per TF at separate single motifs at LPS-opened/Nur77-closed sites in RAW-Ctrl after stimulation versus before LPS stimulation (light grey) or RAW-Nur77 versus RAW-Ctrl both after LPS stimulation (dark grey). Violin plots indicate the median (center line), upper (third quartile; top white box), lower (first quartile; bottom white box) quartile range (box limits), and the minimum/maximum range (vertical lines).

E: Heatmap depicting the expression of members of the AP-1 family that are expressed in BMDM-WT and BMDM-Nur77-KO macrophages after 1 hour of LPS stimulation. Color scale indicates gene expression (z-score).

F: Representative example snapshots of Nur77 ChIP-seq (yellow) at the 5’-end of the Atf3 gene. Y-axis indicates the sequencing signal in fragments per kB per million reads mapped. Nur77 motifs (NBRE) are depicted as red lines.

Supplemental Table S1

LPS-induced, Nur77 repressed genes in RAW cells after 1 and 5 hrs of LPS
Details of the Venn diagrams in Figs. 1C and D.

LPS-induced/ Nur77 repressed 1h LPS
0610040F04Rik
2500002B13Rik
Adamts1
Adm
Arg2
Atf3
Bhlhe40
Ccl2
Ccl7
Cish
Csf1
Dusp1
E230016M11Rik
Eda2r
Egr1
Fabp3
Flrt3
Frmd6
Fst
Gadd45a
Gbp6
Gdf15
Gm14023
Gprc5a
Hbegf
Hilpda
Hspa1b
Ifrd1
Il1a
Il1rn
Il6
Irf1
Klf2
Lif
Lrrc32
Plet1
Plk2
Rhob
Rsad2
Sele
Tcim
Tgm2
Tnfaip2
Tnfrsf1b
Tsc22d1

LPS-induced/ Nur77 repressed 5h LPS		
2500002B13Rik	Fam219a	Mt2
9130230L23Rik	Fam219aos	Mxd1
Abcg2	Fam241a	Mylk2
Acp5	Flrt3	Nckap1
Adamts1	Frmd6	Nos2
Adamts4	Fst	Nr4a3
Adm	Garin1a	Pdgfa
Adora2a	Gbp2	Pdpn
Agrn	Gbp2b	Pdzk1ip1
Ankdd1a	Gbp5	Pik3cb
Apbb2	Gbp7	Plet1
Aqp9	Gdap10	Plpp3
Arg2	Gdf15	Ppfia3
Arid5b	Gem	Prdm9
Atf3	Gipr	Prkg2
Atp13a4	Glipr2	Ptafr
Bambi-ps1	Gm14023	Ptger4
Batf	Gm2011	Ptges
Bcar1	Gprc5a	Rab32
Bcl3	Gramd2b	Rapgef5
C9orf72	H2-M2	Rasa4
Camk2d	Hbegf	Rps6ka2
Ccbe1	Hcar2	Rsad2
Ccl17	Hck	Saa3
Ccl2	Heatr9	Serpine1
Ccl7	Hilpda	Slc28a2
Ccrl2	Icosl	Slc28a2b
Cd274	Igsf3	Slc39a2
Ceacam16	Il13ra2	Slc49a3
Chst11	Il15	Slc6a12
Clcn7	Il1rn	Slc7a2
Clec2d	Il20rb	Slpi
Cmpk2	Il33	Spink5
Crybg2	Irf1	Src
Csf1	Jund	Stat5a
Csf3r	Klk8	Syt7
Dcstamp	Klk9	Timp1
Ddx60	Ldlrad4	Tmem202
Dgat2	Lif	Tnfaip2
Dpf1	Lpl	Tnfrsf14
Dusp1	Lrrc32	Tnfrsf1b
Eda2r	Mamld1	Tnfrsf8
Eeig2	Mapre2	Tnfrsf9
Eno2	Marcks	Trpm4
Ero1a	Mcoln2	Tsc22d1
Eva1b	Mir6381	Ubxn2a
Exoc3l4	Mitf	Upp1
Ext1	Mmp9	Urah
Fabp3	Msantd3	Wnt6

Supplemental Table S1: Additional data to Venn diagrams in Figure 1C-D.
List of LPS-induced/Nur77-repessed genes at 1 hour (left column) and 5 hours of LPS stimulation (3 right columns). Highlighted genes also show enhanced expression in LPS-treated Nur77-KO BMDMs compared to LPS-treated WT BMDMs (LPS treatment for 1, 5, or 18 hours).

Supplementary Table S2

NBRE, NurRE and DR5 motifs in Nur77 ChIP-seq peaks of genes
Details of the Euler diagram in Fig. 2C.

**NBRE motif only**	**NBRE + AP1 motif**	**AP1 motif only**	**Other**
Bcar1	0610040F04Rik	Adamts1	Acp5
Camk2d	Adora2a	Adm	Atf3
Pdzk1ip1	Ankdd1a	Arid5b	Csf1
Rab32	Apbb2	Batf	Egr1
Rsad2	Arg2	Bcl3	Gbp5
Ubxn2a	Ccl2	Ccbe1	Gbp7
Wnt6	Chst11	Ccl17	Hbegf
	Cish	Cd274	Hck
	Dusp1	Ceacam16	Il15
	Eda2r	Clcn7	Mcoln2
	Ext1	Dcstamp	Src
	Flrt3	Dgat2	Syt7
	Gadd45a	Eva1b	
	Icosl	Fam241a	
	Igsf3	Frmd6	
	Il1a	Fst	
	Lrrc32	Gbp2	
	Marcks	Gbp6	
	Nr4a3	Gdf15	
	Pik3cb	Gprc5a	
	Plet1	Hcar2	
	Plk2	Il20rb	
	Plpp3	Il6	
	Prkg2	Jund	
	Ptges	Klf2	
	Rps6ka2	Ldlrad4	
	Slc6a12	Lpl	
	Tcim	Mapre2	
	Tnfrsf1b	Mitf	
	Tnfrsf8	Mmp9	
		Msantd3	
		Mt2	
		Mylk2	
NurRE		Nos2	
		Pdgfa	
DR5		Pdpn	
		Ptafr	
		Ptger4	
		Rapgef5	
		Rasa4	
		Rhob	
		Saa3	
		Serpine1	
		Slc39a2	
		Slpi	
		Spink5	
		Tgm2	
		Tnfaip2	
		Tnfrsf14	

Supplemental Table 2: Additional data to the Euler diagram in Figure 2C for genes with an annotated peak in the HA-Nur77 ChIP-seq. Different groups of genes are indicated either with an annotated peak containing an NBRE-motif only (first column), an NBRE- and AP-1 motif in the same peak (second column), an AP-1 motif only (third column), or genes without any of these motifs (last column). In addition, genes with annotated peaks containing NurRE motifs are indicated (light green) and one gene with a DR5-motif (dark green) was identified.
